# A Review of Oxygen Carrier Materials and Related Thermochemical Redox Processes for Concentrating Solar Thermal Applications

**DOI:** 10.3390/ma16093582

**Published:** 2023-05-07

**Authors:** Stéphane Abanades

**Affiliations:** Processes, Materials and Solar Energy Laboratory, PROMES-CNRS, 7 Rue du Four Solaire, 66120 Font-Romeu-Odeillo-Via, France; stephane.abanades@promes.cnrs.fr

**Keywords:** metal oxide, solar fuels, hydrogen production, water splitting, CO_2_ conversion, ammonia synthesis, heat storage, thermochemical redox reactions, oxygen separation, solar chemical processing

## Abstract

Redox materials have been investigated for various thermochemical processing applications including solar fuel production (hydrogen, syngas), ammonia synthesis, thermochemical energy storage, and air separation/oxygen pumping, while involving concentrated solar energy as the high-temperature process heat source for solid–gas reactions. Accordingly, these materials can be processed in two-step redox cycles for thermochemical fuel production from H_2_O and CO_2_ splitting. In such cycles, the metal oxide is first thermally reduced when heated under concentrated solar energy. Then, the reduced material is re-oxidized with either H_2_O or CO_2_ to produce H_2_ or CO. The mixture forms syngas that can be used for the synthesis of various hydrocarbon fuels. An alternative process involves redox systems of metal oxides/nitrides for ammonia synthesis from N_2_ and H_2_O based on chemical looping cycles. A metal nitride reacts with steam to form ammonia and the corresponding metal oxide. The latter is then recycled in a nitridation reaction with N_2_ and a reducer. In another process, redox systems can be processed in reversible endothermal/exothermal reactions for solar thermochemical energy storage at high temperature. The reduction corresponds to the heat charge while the reverse oxidation with air leads to the heat discharge for supplying process heat to a downstream process. Similar reversible redox reactions can finally be used for oxygen separation from air, which results in separate flows of O_2_ and N_2_ that can be both valorized, or thermochemical oxygen pumping to absorb residual oxygen. This review deals with the different redox materials involving stoichiometric or non-stoichiometric materials applied to solar fuel production (H_2_, syngas, ammonia), thermochemical energy storage, and thermochemical air separation or gas purification. The most relevant chemical looping reactions and the best performing materials acting as the oxygen carriers are identified and described, as well as the chemical reactors suitable for solar energy absorption, conversion, and storage.

## 1. Introduction

Redox cycles involving metal oxides as oxygen carrier materials have been extensively used for many years in chemical-looping technologies such as combustion (CLC), as well as reforming (CLR) or gasification (CLG) processes [1,2,3,4]. Typically, the oxide is used as a solid oxygen carrier; it is chemically reduced using a fuel and then re-oxidized separately in air, which facilitates the capture of CO_2_ because the stream from the fuel reactor predominately contains CO_2_ and H_2_O. Indeed, the separation of the fuel from air simplifies the number of chemical reactions in combustion and leads to a cleaner (no NO_x_) and lower-temperature fuel combustion. This provides CLC noteworthy benefits in comparison with competing carbon capture technologies generally involving a significant energy penalty associated with either post-combustion scrubbing systems or work input required for air separation plants (oxy-combustion systems). Similarly, CLR and CLG can be used for hydrogen or syngas production from natural gas, coal, or biomass without the need for catalysts, and solar-driven chemical-looping processes have been adapted and developed [5,6,7,8,9,10,11,12,13,14,15,16,17,18,19,20]. One challenge in chemical-looping processes is maintaining the oxygen carrier stability over many cycles.

Different solar thermochemical processes derived from chemical looping have been considered. Solar energy, although intermittent and dilute, can be concentrated to produce high-temperature heat as a process energy source. This energy can be stored long term in the form of transportable fuels or chemical commodities. In addition, the development of heat storage technologies is also a strong benefit to overcome the solar energy intermittency and to allow for a continuous solar thermal process operation (the possible storage of solar heat is strongly advantageous in comparison with photovoltaics). Various applications reliant on the conversion of concentrated solar energy can benefit from the attractive redox properties of oxide materials. Two-step thermochemical processes based on oxygen carrier materials are developed as a suitable means to store and convert solar energy in the form of fuels (H_2_, syngas, ammonia), thermochemical heat, or O_2_ and N_2_ streams. One of the most well-known processes is based on a redox material, which is heated under low-oxygen partial pressure for reduction and oxygen release as a first step. The oxide is then re-oxidized exothermically in a second step at a lower temperature, with steam or carbon dioxide as an oxidant to generate hydrogen or carbon monoxide. The heat required to drive the endothermic reaction at high temperature can be supplied by concentrated solar energy, allowing the conversion and storage of solar energy into solar fuels with high energy density. To synthesize liquid fuels, the as-produced syngas can be further processed to hydrocarbons such as kerosene, diesel, or gasoline in the Fischer–Tropsch process or to methanol. The renewable hydrogen can also be used in combination with nitrogen to form green ammonia in the Haber–Bosch process. Another new looping route makes use of metal oxide/nitride two-step cycles for the direct conversion of H_2_O steam into ammonia with the supply of nitrogen.

**Figure 1:**
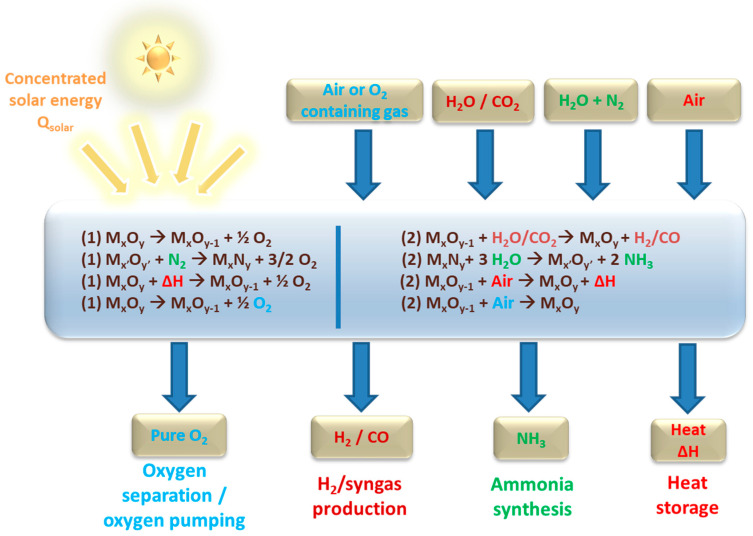
Metal oxide redox cycles applied to solar chemical processing involving thermochemical oxygen separation/oxygen pumping, fuel production (H_2_, CO), ammonia synthesis, and thermochemical energy storage.

In addition, other possible oxidants for the low temperature step include air (which can be separated into oxygen and a stream of nitrogen), but also O_2_-containing inert gases for their purification by oxygen extraction (which is a suitable path for inert gas recycling or for oxygen pumping from inert gas streams to lower the O_2_ partial pressure, thereby favoring the first step of redox cycles). Instead of exploiting the oxygen storage capacity of the materials, the thermal effect of reversible redox reactions can also be valorized, which allows thermochemical energy storage for cyclical heat charge/discharge, paving the way to continuous solar energy supply on demand. These thermochemical routes are summarized in Figure 1.

Screening and identifying suitable metal oxides with superior and long-term stable redox performance, manufacturing and shaping porous ceramic structures with enhanced heat and mass transfer properties, and the design/engineering of robust and scalable solar reactors yielding fuels with high rate, selectivity, and efficiency are the main challenges in the field. Moreover, delivering concentrated solar process heat at elevated temperatures necessitates coupling solar receivers with high-flux solar concentrating optics (developed mainly for solar thermal electricity generation via concentrating solar power, CSP [21]). The effective development of solar thermochemical processes is thus closely tied to the advances in CSP systems and their scalability. Solar field design is carried out to determine the suitable size and performance of heliostat fields capable of delivering power to the receiver at a given solar concentration ratio. A central tower receiver system is a relevant practical solar concentrating technology able to deliver large power levels to a solar chemical reactor [22]. The present status and future prospects of solar thermochemical fuels show potential commercial viability in the near future [23,24]. Techno-economic studies and life-cycle analyses of large-scale thermochemical plants have also confirmed the environmental benefits and favorable cost prospects of such solar technologies [25,26,27,28,29,30,31,32,33].

The range of investigated materials for the different applications is very broad, especially in the field of solar fuels from H_2_O and CO_2_ feedstocks. The redox materials can be classified as either (i) volatile/non-volatile oxides, or (ii) stoichiometric/non-stoichiometric oxides, depending on the nature of the involved redox reactions. Some oxides indeed feature discrete (stoichiometric) transitions in the metal oxidation state (such as ZnO/Zn, SnO_2_/SnO, or Fe_3_O_4_/FeO) accompanied by a phase change (solid-to-gas or solid-to-liquid) during the reduction step. Others feature a continuous redox activity with the continuous release/uptake of oxygen depending on both the temperature and oxygen partial pressure, thanks to oxygen vacancies (non-stoichiometry) that facilitate ionic oxygen diffusion in the solid state (no crystalline phase change occurs during redox cycles, as in the case of ceria and perovskite systems).

The oxides undergoing a stoichiometric phase change during reduction, typically simple oxides such as ZnO or Co_3_O_4_, exhibit a much larger oxygen exchange capacity and specific energy storage than the materials undergoing partial reduction (non-stoichiometric) such as ceria and perovskites. In contrast, the materials featuring partial reduction generally show faster redox kinetics and higher activity at low temperature. The selection of candidate materials for the various applications is often a trade-off between a high specific energy storage (or oxygen exchange capacity), fast kinetics, and low operation temperature [34].

It is important to note that the suitable materials for each application do not overlap, and universal materials adapted for any kind of application do not exist. In practice, the materials suitable for solar fuel production (H_2_O and CO_2_ splitting) have no relevant properties for thermochemical energy storage or oxygen separation, and vice versa. Generally, the materials able to split H_2_O or CO_2_ (e.g., CeO_2−δ_) require a too-high reduction temperature for practical application in energy storage/oxygen storage. Inversely, the materials with a high oxygen or energy storage capacity (e.g., Co_3_O_4_) under mild operating conditions (i.e., moderate temperature or high-oxygen partial pressure) are not able to split H_2_O or CO_2_. Materials that can be easily reduced (in air) have a low activity toward H_2_O or CO_2_ splitting. Conversely, the active materials for the splitting step are difficult to reduce and display a high reduction temperature for oxygen release (strong oxygen affinity). 

Regarding the influence of the oxidant, all of the reduced materials are able to re-oxidize spontaneously in air, but a more restrained number is able to be oxidized by H_2_O or CO_2_ according to their thermodynamic properties (the redox behavior is globally ruled by the Gibbs free enthalpy change ΔG°). Re-oxidation with O_2_ can be simply carried out by decreasing the temperature (via temperature swing) at a constant oxygen partial pressure or by increasing the oxygen partial pressure (via pressure swing) at a constant temperature, regardless of the oxide material.

Numerous experimental studies have focused on the materials’ reactivity characterization for the different considered thermochemical applications. In particular, with the recently growing interest in non-stoichiometric materials (ceria-based or perovskite oxides) and the large number of possible dopants for incorporation, many studies have been performed, but the comparison of material performance is tricky due to the different synthesis methods, material morphologies, experimental procedures for reactivity characterization, and applied cycling conditions. Moreover, most experimental studies generally focus on a given material formulation without any inter-comparison between different classes of materials, which makes difficult to appreciate the real superior redox activity often claimed in such studies. In addition to empirical methods based on material synthesis and characterization, computational methods such as density functional theory (DFT) calculations have recently gained interest to allow for the prediction of material properties [35]. This can help in the rapid screening of large sets of materials for target properties, but does not replace experimental testing to assess their real redox activity.

This review particularly highlights the recent efforts in materials science for solar thermochemical processes that avoid the use of carbonaceous feedstock as reagents. The solar thermochemical conversion processes of methane, coal, or biomass (cracking, reforming, gasification technologies [36,37,38,39,40]) will not be discussed. The next sections summarize the current knowledge and research gaps for the different thermochemical applications, including: (i) solar thermochemical fuel generation from water/CO_2_-splitting redox processes, (ii) ammonia synthesis based on chemical looping cycles, (iii) solar thermochemical energy storage for off-sun continuous processing, and (iv) thermochemical air separation/oxygen pumping for gas purification, while focusing on the latest advances in the research field of redox materials and related solar thermochemical processes. 

## 2. Solar Thermochemical Fuels from Two-Step H_2_O and CO_2_-Splitting Redox Cycles

One of the most developed solar chemical processes aims to produce solar fuels from only H_2_O and CO_2_ feedstocks. The considered approach is to apply thermochemical two-step redox cycles, encompassing the solar-driven reduction of a metal oxide followed by its re-oxidation with H_2_O and/or CO_2_ to generate H_2_ and/or CO (syngas), as the building blocks for a wide variety of synthetic drop-in fuels such as methanol, diesel, gasoline, and kerosene. CO_2_ conversion to fuels is an attractive pathway for CO_2_ utilization. Using CO_2_ emitted and captured from industrial gases or directly captured from air (direct air capture) as a commodity to be transformed into a solar energy carrier offers a sustainable alternative to CO_2_ sequestration (CCS). This is equivalent to reversing the combustion process with sunlight to provide a sustainable path towards powering the transportation sector with carbon-neutral synthetic fuels that are compatible with transportation infrastructure [23]. 

**Figure 2:**
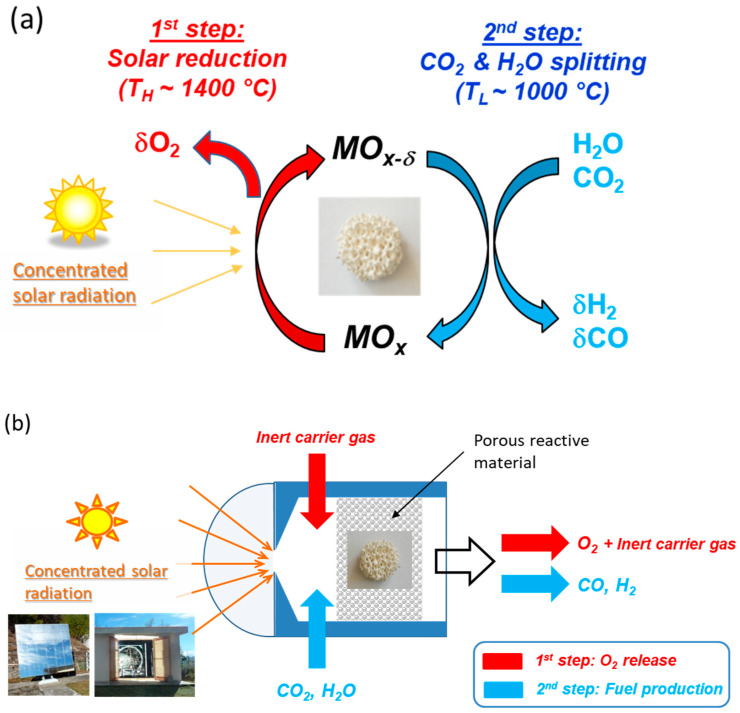
Solar fuel production from H_2_O and CO_2_ redox splitting: (**a**) two-step solar-driven thermochemical H_2_O and CO_2_-splitting cycles, (**b**) operating principle of monolithic solar reactors integrating a porous reactive structure with a first reduction step in inert gas and a second oxidation step with H_2_O or CO_2_.

Two-step cycles, as shown in Figure 2a, involve redox pairs to dissociate water (or CO_2_) molecules into separate streams of H_2_ (or CO) and O_2_ [41,42,43,44,45,46,47]. Such cycles are composed of a first activation step, consisting of oxide reduction (Equation (1)), followed by an oxidation step leading to H_2_ (or CO) release (Equation (2)).

M_x_O_y_ → M_x_O_y−δ_ + δ/2 O_2_ (1)

M_x_O_y−δ_ + δH_2_O (CO_2_) → M_x_O_y_ + δH_2_ (CO) (2)

The first endothermal step requires thermal energy supplied by concentrated solar radiations. This reaction occurs at a significantly lower temperature than the direct thermolysis of H_2_O or CO_2_ (requiring temperatures above 2500 °C), but still requires temperatures of about 1400 °C achievable with concentrated solar energy. The second exothermal step does not theoretically require any energy input. Several redox pairs have been identified and investigated, such as ZnO/Zn [48,49,50,51], SnO_2_/SnO [52,53,54,55], Fe_2_O_3_/FeO [56,57,58], MFe_2_O_4_/MFe_2_O_4−δ_ [59,60], CeO_2_/CeO_2−δ_ [61,62,63,64,65,66], and perovskites (ABO_3_/ABO_3−δ_) [67,68,69,70,71,72]. Alternatively, solid or gaseous carbonaceous reductants (C, CH_4_) can be used to lower the temperature of the reduction step, as shown for, e.g., zinc, magnesium, tin, iron, or tungsten metal production [73,74,75,76,77,78,79,80]. Metal oxide redox pairs must be reactive enough to produce a significant amount of fuel during each cycle and be stable under long-term cycling to avoid any performance degradation. Zinc- and tin-based cycles offer a high fuel productivity and reactivity with H_2_O or CO_2_ [81,82,83], but suffer from low vapor pressure of the reduced species, producing gaseous compounds that are difficult to recover without any recombination reaction during the reduction step [49]. For such volatile oxide cycles, decoupled reactors must be used, i.e., a reduction step carried out in a first reactor heated by solar energy and an oxidation step in a second reactor with particle circulation between both reactors. For non-stoichiometric oxide cycles, a single reactor can be used by alternating the temperature with the reactive solid remaining in the reactor in the solid state during both cycle steps, as illustrated in Figure 2b. The reacting material can be either in the form of particles (e.g., packed or fluidized beds) or structured with a porous architecture, both aiming to offer a large solid–gas interface for reactions and to favor heat and mass transfer.

The iron oxide cycle shows strong deactivation during cycling due to the melting or sintering at the reduction temperature [84]. To overpass the deactivation of the iron oxide cycle due to sintering, doping with other metals was studied to improve cyclability and decrease the reduction temperature [85]. Ferrites (MFe_2_O_4_) were proven to be capable for partial reduction (non-stoichiometric reduced phase) at lower temperatures than iron oxide, and still active for water-splitting reactions [60,86,87,88,89,90,91]. Ferrites such as NiFe_2_O_4_, CoFe_2_O_4_, and ZnFe_2_O_4_ have been considered and show good performance in thermochemical hydrogen production, but they still suffer from deactivation after several cycles. To alleviate this deactivation due to the high temperature reduction (1300–1400 °C), ferrites were coated onto ceramic supports (such as SiC honeycomb structures, ZrO_2^−^_ or yttria-stabilized zirconia particles) [92,93,94]. Although the material stability is improved, the fuel yield is impacted by the support mass and is not high enough to reach high conversion yields [95,96]. 

Material performance is strongly ruled by thermodynamics, highlighting the importance of the enthalpy and entropy change during oxygen exchange [97]. To improve the performance of the reference ceria cycle, new materials should exhibit larger entropy and lower enthalpy changes to decrease the temperature of the reduction step, but there is a compromise with the thermodynamic driving force for oxidation (splitting step) that results in less efficient materials overall. Among the most promising redox pairs for thermochemical water splitting, non-volatile and non-stoichiometric oxides (ferrites, ceria, perovskites) have opened up a new direction for the development of solar thermochemical materials due to their favorable thermo-kinetic properties, their moderate reduction temperature, and excellent structural stability [44]. However, the solar-to-fuel efficiency suffers from low conversion rates and the material reduction yield still needs to be improved, as well as their oxidation rates. A critical analysis of redox materials for solar thermochemical conversion was conducted based on thermodynamic considerations according to their applications [34]. Regarding solar fuel production, the conclusion is the well-known compromise between a low reduction enthalpy to decrease the reduction temperature, and a high enough entropy to keep a driving force favoring good kinetics during the oxidation step. Maintaining high splitting favorability while lowering the material’s reduction temperature is targeted. The general trend in this domain reveals that as the material becomes easier to reduce, it is less likely to split water (or CO_2_) [98]. The efforts undertaken to optimize the performance of ceria and perovskite redox cycles are summarized below.

### 2.1. Ceria-Based Cycles

**Figure 3:**
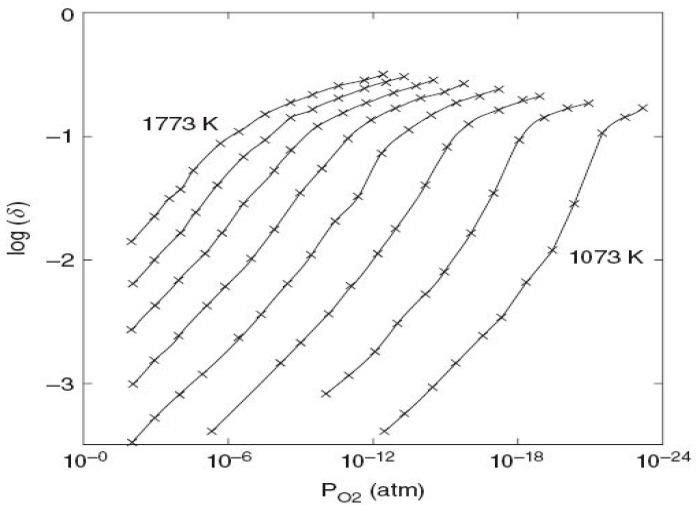
Isothermal plots of δ as a function of p_O_2__ for CeO_2_/CeO_2−δ_ showing an increase in δ when increasing the temperature or decreasing the pressure [100].

Ceria, as redox material for two-step water-splitting thermochemical cycles, was first investigated in 2006 [99]. Cerium (IV) oxide can be partially reduced to Ce (III) upon heating (~1400 °C) with continuous oxygen vacancy formation during thermal reduction (without any reductant), and has the ability to keep its fluorite crystal structure for large sub-stoichiometries (δ in CeO_2−δ_), while the reduced Ce^3+^ species are highly reactive for the splitting step. Ceria exhibits rapid kinetics and crystallographic stability. The reversible and fast transition between Ce^3+^ and Ce^4+^ in redox cycling was largely employed in catalytic converters for the control of emissions from engine vehicles. The reduction extent δ (oxygen content in ceria or oxygen non-stoichiometry) is directly correlated to the temperature and the oxygen partial pressure, as illustrated in Figure 3 [66,100,101,102]. The higher the temperature or the lower the oxygen partial pressure, the higher the reduction extent. Note that a reducing agent (such as CH_4_) can be alternatively used to lower the reduction temperature of ceria typically below 1000 °C and the process involves the partial oxidation of methane with simultaneous ceria reduction (chemical-looping reforming [103,104,105]).

The main drawback of the cerium oxide cycle is the limited fuel amount produced per cycle, which is directly correlated to the low reduction extent δ. A limited reduction yield is obtained by heating ceria at 1500 °C under an inert gas flow with an oxygen non-stoichiometry δ of 0.04–0.06 that corresponds to about 10% of the theoretical maximum reduction yield [66], which results in a limited solar-to-fuel efficiency (5.25% in [106]). In terms of thermodynamic properties, ceria has a high reduction enthalpy that limits the reduction extent; conversely, it favors the driving force for the oxidation step, inducing high reactivity with oxidants and thereby excellent kinetics. To enhance the low reduction extent, several approaches have been investigated, including doping with another metal and improving the material morphology and microstructure [64,107].

Dopant incorporation into the ceria structure has been widely investigated to improve the fuel production yield [108,109,110,111]. Several metals such as Zr, Fe, Gd, Y, Sm, Hf, Mn, Ni, Cu, Ti, Pr, Ta, and La were incorporated in the fluorite ceria structure in different amounts and tested during thermochemical cycles. As a conclusion of these doping investigations, the reduction enthalpy could be decreased by partially substituting Ce^4+^ with another cation favoring oxygen vacancy creation. This led to an improved reduction extent at the expense of lower water-splitting activity. However, ceria doping with Zr, Zr/Gd, and Pr/Zr was reported to improve thermochemical hydrogen production [112] with an O_2_ and H_2_ production increase of 30% and 11%, respectively, with Zr insertion (ternary compounds are detrimental to H_2_ production). The study also dealt with kinetic analysis that revealed a surface rate controlling mechanism. DFT calculations highlighted that the surface H_2_ formation step is rate limiting, having greater activation barriers than oxygen bulk diffusion.

The material morphology also plays an important role in improving the performance of ceria during thermochemical cycles and can enhance the solar-to-fuel efficiency. On the one hand, as the reduction step is governed by diffusion mechanisms [113] and limited by heat transfer [62], an optimized morphology reducing the diffusion length can improve heat and mass transfer, and increase the reduction yield. On the other hand, since the oxidation step is a solid/gas reaction that is rate limited by surface mechanisms, an increase in the surface area positively affects the kinetics. Several material shaping options were tested to improve the reactivity of ceria. 

Ceria-based ceramics with designed morphologies and microstructures were reviewed [107], highlighting an influence of the porosity and the microstructure on reactivity. Ceria powders, fibers, felts, reticulated porous structures, and 3D-ordered macroporous (3DOM) materials have been studied, showing an increased amount of fuel produced compared to non-designed ceria powder. The main methodology used to synthesize reticulated or ordered ceramics is the replication method on polymer scaffolds. The reactivity of such ceramic foams was investigated under real concentrated solar flux [114,115,116]. A maximum CO production rate approaching 10 mL/min/g was achieved with ceria reticulated foams (composed of µm-sized grains forming an interconnected macroporous network within the struts) during their re-oxidation upon free cooling with a pure stream of CO_2_ (after a reduction step at 1400 °C) [65], thus noticeably outperforming (by a factor of about ×8) the maximum values reported to date. This performance was attributed to the fine and stable granular microstructure of the reticulated ceria foams. The solar reactor reliability and robustness during high-temperature two-step redox cycling were demonstrated with an average cycle production of 5.1 mL/g of H_2_ and CO, and peak solar-to-fuel efficiencies over 8%. The monolithic solar reactor was further implemented to characterize reticulated porous foams with controlled cell sizes (10–60 ppi, pores per inch), stacked to form a porosity gradient enabling efficient volumetric solar radiation absorption [116]. An increase in the fuel production rate by tuning the operating parameters confirmed the importance of material shaping for integration in solar thermochemical reactors. 

On the basis of the prior experience from solar gas turbine systems, a combined receiver/reactor was designed and built, operating at temperatures between 1400 °C and 1000 °C using radiative power of up to 150 kW generated by a large-scale solar simulator [117]. The reactor with a total volume of 90 L contained an open porous ceramic foam structure made of 10 ppi zirconium oxide (ZrO_2_, 166.1 kg) reticulated porous ceramic (RPC) foams coated with cerium oxide (CeO_2_, 44.8 kg) acting as the redox material, and it produced up to 8.8 g of hydrogen per cycle (i.e., 98 µmol/g_ceria_).

**Figure 4:**
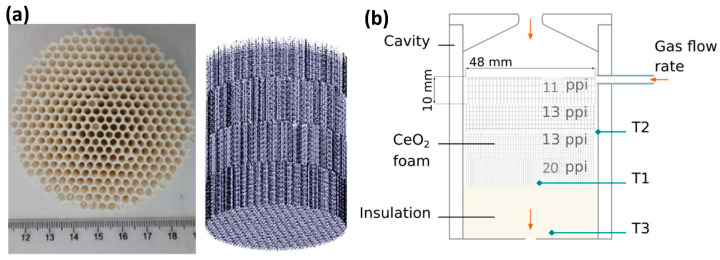
Example of (**a**) hierarchically ordered porous structures obtained from 3D-additive manufacturing followed by the replication method and (**b**) scheme of the hierarchically ordered foam structures stacked in a cavity to form a vertical porosity gradient for enhanced solar radiation absorption [114].

Reticulated porous structures of ceria-based mixed oxides were prepared to overpass the low cyclability of such mixed oxides in powder form [118]. Ce_0.9_Fe_0.1_O_2_ showed the best results regarding H_2_ production and cyclability. Mn-doped ceria was synthesized and then coated on MPSZ ceramic foam at 30% mass for solar testing in thermochemical cycles [119], showing an improved O_2_ and H_2_ release compared to pristine ceria coated on ceramic foam, despite a strong deactivation after several cycles and the low stability of the material. Ordered ceria structures prepared by the replication of optimized scaffolds obtained from additive manufacturing or robocasting were considered [120]. The design of hierarchically ordered structures with porosity gradient was performed [121] for higher radiation penetration compared to reticulated structures with uniform porosity. The reduction step showed a higher amount of oxygen released because of the higher heating rate and more uniform temperature distribution. Additive manufacturing to synthesize an ordered porous ceria structure via the replication method was also investigated [114]. Honeycomb-type channeled structures with different porosities were synthesized and stacked in a cavity reactor to obtain a porosity gradient (Figure 4). An improvement in the reduction yield was measured during on-sun experiments, confirming that enhanced heat and mass transfer is beneficial to increase the reduction extent. On the other hand, the oxidation step was not improved, as it implies surface reaction mechanisms. 

**Figure 5:**
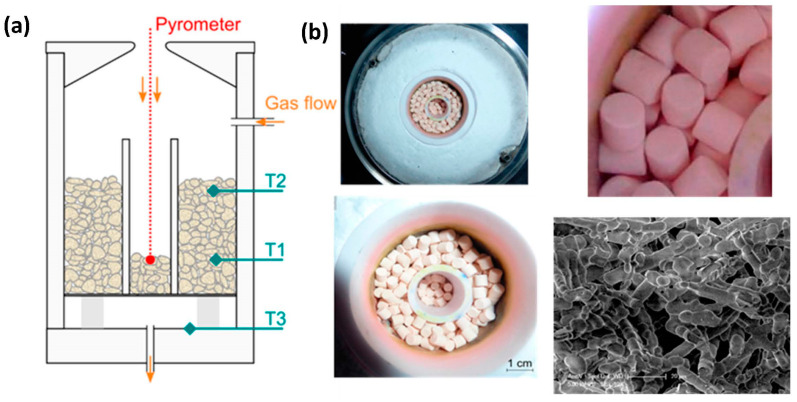
Example of customized ceria materials used in fuel generation from water/CO_2_-splitting redox processes [123]: (**a**) scheme of the packed-bed cavity-type solar reactor and (**b**) fibrous ceria pellets inserted in the reactor cavity, along with their morphology.

**Figure 6:**
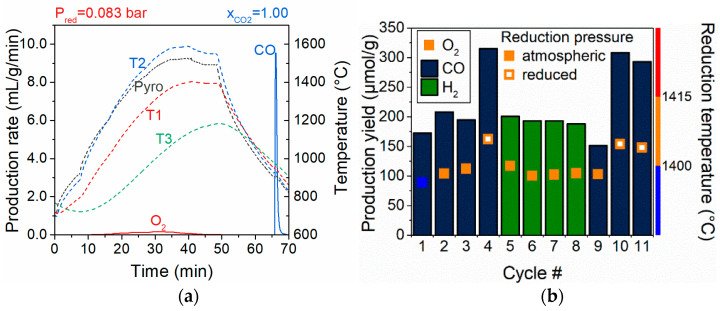
Reactivity of fibrous ceria pellets in packed-bed solar reactor during consecutive cycles [123]: (**a**) O_2_ and CO production rate profiles during a CO_2_-splitting cycle with peak production rate of 9.5 mL/min/g and (**b**) gas production yields during successive thermochemical CO_2_ and H_2_O-splitting cycles performed in a solar reactor at different pressures during the reduction step (squares represent the O_2_ production amount, bars refer to the CO or H_2_ fuel production, and the reduction temperature is indicated by the color of the square).

Additional work has been conducted on tuning the ceria microstructure to improve the reactivity of the splitting step, which is governed by a surface reaction mechanism. For instance, ceria porous microspheres were synthesized and tested during thermochemical cycles [122], as well as fibrous ceria pellets (Figure 5) [123] and cork-templated ceria (3DOM ecoceramics) [124,125,126]. The reactivity of these materials was investigated in packed-bed solar reactors during consecutive cycles (Figure 6), showing an improved reactivity compared to ceria powders.

### 2.2. Perovskite-Based Cycles

Thermodynamics, design principles, and experimental results on perovskite-based cycles have been extensively reported [67,68,70,127,128]. Perovskites can accommodate a huge composition space, are thermally stable, and can accept oxygen non-stoichiometry without any phase changing. From the wide composition possibilities, computational calculations and experimental screening studies have permitted to identify materials based on lanthanum–manganese, lanthanum–cobalt, and yttrium–manganese associated with other dopants as the most promising formulations for solar thermochemical cycles. They allow a decrease in the reduction temperature compared to ceria, in spite of a lower reactivity for the splitting step. Perovskites also need a high steam or CO_2_ excess (and a large temperature swing) to drive re-oxidation to completion, which negatively impacts the solar-to-fuel efficiency. Based on a comparison between perovskites and ceria for solar thermochemical H_2_O and CO_2_ splitting [129], the solar-to-fuel conversion efficiency of Zr-doped ceria or pure ceria is higher than for currently known perovskites. This is explained by the unfavorable large temperature swing necessary for perovskites between the two steps and the high oxidant flowrate required to increase the reactivity of the splitting step. However, the influence of the temperature swing on the overall efficiency can be omitted because the solar field design is based on the high-temperature reduction step, and a large temperature gap between steps should have no influence on it. The energy necessary to heat the active material from the oxidation temperature to the reduction temperature is provided by the existing solar field and should not induce additional costs. The main drawback of a large temperature gap between the two steps is the time necessary to reach the reduction temperature, which could reduce the number of cycles per day and negatively impact the annual fuel production. 

**Figure 7:**
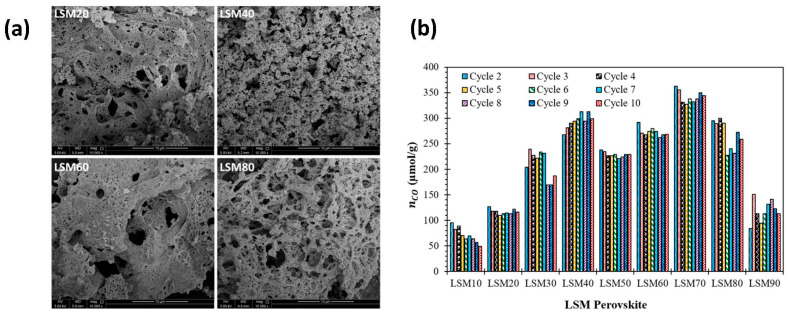
La/Sr/Mn perovskites synthesized by solution combustion: (**a**) porous morphologies characterized by SEM and (**b**) CO production yield during 10 consecutive cycles [130].

The combustion synthesis of La_1−x_Sr_x_MnO_3_ perovskites was performed to assess their CO production during 10 consecutive thermochemical cycles [130], resulting in a porous powder morphology and yielding 342.1 µmol_CO_/g for La_0.3_Sr_0.7_MnO_3_ (LSM70) with a thermal reduction at 1400 °C for one hour and an oxidation step at 1000 °C for 30 min with a 50% CO_2_ gas mixture. The material showed good thermal stability and a stable reaction yield during cycling (Figure 7). A study on La_0.4_Sr_0.6_Mn_1−x_Al_x_O_3_ (x = 0.4, 0.5, and 0.6) perovskite oxides between 1400 °C for the reduction and 800 °C for the oxidation reaction revealed the highest H_2_ production of 144 µmol/g for x = 0.6 [131]. Another study revealed a production of CO of 114 µmol g^−1^ with La_0.6_Sr_0.4_Mn_0.6_Al_0.4_O_3_ [132]. The solid-state re-oxidation kinetics of A/B-site substituted LaMnO_3_ during thermochemical CO_2_ conversion were studied, showing that the re-oxidation mechanism varied depending on the composition of the oxygen carrier [133]. Correlations between the lattice geometric parameters of perovskite oxides and thermochemical redox CO_2_-splitting performance were also highlighted [134,135].

Reticulated porous lanthanum strontium manganite structures were tested for solar thermochemical hydrogen production in 50 cycles at 1400 °C, producing ca. 200 µmol/g of H_2_ per cycle [136].

New perovskites with increased performance for thermochemical fuel production were proposed, such as perovskites including reducible cerium atoms in the structure. A Ce_x_Sr_2−x_MnO_4_ Ruddlesden–Popper layered perovskite phase was considered [137,138], in which cerium atoms occupy the A-site, and tunable cerium amounts from x = 0.1 to 0.3 were synthesized. Ce_0.2_Sr_1.8_MnO_4_ was identified as the most interesting composition, yielding a higher amount of H_2_ compared to ceria even with a relatively low steam-to-hydrogen ratio. In another study [139], Ca_0.5_Ce_0.5_MnO_3_ was identified as a good candidate for thermochemical water splitting. The authors performed a theoretical study on the Ca-Ce-M-O (M = 3d transition metal) composition space with the aim to discover a phase with an increased reduction entropy because of simultaneous A-site and B-site reduction, which needs to be demonstrated experimentally. Another cerium-based perovskite related to the lanthanum–strontium manganite well-known phase was assessed [140]. A thermogravimetric analysis over 100 consecutive CO_2_-splitting cycles demonstrated an improved conversion extent compared to ceria and known perovskites. The chemical composition was La_0.48_Sr_0.52_(Ce_0.06_Mn_0.79_)O_2.55_ and a reversible phase change during the redox cycle was evidenced by neutron diffraction. Another cerium-containing phase, CeTi_2_O_6_ with a brannerite structure, was identified as a candidate material for thermochemical water-splitting cycles from DFT calculation to compute the reduction enthalpies (i.e., oxygen vacancy formation energies) [141]. In fact, CeTi_2_O_6_ shows a high reduction entropy and a low reduction enthalpy associated with good thermal stability, but the performances still need to be experimentally demonstrated.

LaCo_0.7_Zr_0.3_O_3_ was also studied as a redox cycling material [142]. A thermodynamic study showed the impact of pumping and inert gas purging to reduce oxygen partial pressure on the solar-to-fuel efficiency. The best conversion yield, obtained with 75% heat recovery at 1300 °C, only reached 1.36% and a very high amount of CO produced per cycle (1400–1700 µmol/g) was reported, which needs to be confirmed. Sr_2_CoNb_0.3_Ti_0.7_O_6_ was reported to exhibit a production of H_2_ as high as 410 µmol/g during eight consecutive cycles [143]. The most surprising fact is that the cycles were realized isothermally at 700 °C. A La_0.6_Ca_0.4_Mn_0.7_Al_0.3_O_3_ material coated on reticulated ceramic foams was studied [144]. SiC foams with different porosities were dip-coated with the perovskite (amount of ca. 2–3%) and the reactivity during CO_2_-splitting cycles was quite low (66 µmol/g with a reduction temperature of 1240 °C and an oxidation temperature of 1050 °C). A new perovskite phase (Gd_0.5_La_0.5_Co_0.5_Fe_0.5_O_3_) for thermochemical water-splitting was identified by computational materials screening and its redox activity was experimentally demonstrated [145], showing a good cycling stability and a higher H_2_ production than ceria (reduction at 1350 °C and hydrolysis at 850 °C), but the H_2_ yield was lower than other perovskites such as BaCe_0.25_Mn_0.75_O_3_ and Sr_0.4_La_0.6_Mn_0.6_Al_0.4_O_3_. Regardless of the H_2_ production performance, the methodology of the computer-aided discovery of new materials is time saving compared to experimental screening because of the wide compositional space of perovskites. Different La_1−x_Sr_x_Mn_1−y_Al_y_O_3_ material compositions were investigated with synchronized Sr/Al or Sr/Mn substitution and the substitution of Al by Cr [146]. La_0.7_Sr_0.3_Mn_0.9_Cr_0.1_O_3_ and La_0.7_Sr_0.3_Mn_0.8_Cr_0.2_O_3_ yielded the best O_2_ and H_2_ average amounts produced. The thermodynamic solar-to-fuel performance of chromium-doped perovskite La_0.6_Sr_0.4_Mn_1−y_Cr_y_O_3_ was assessed [147]. Two advantages of substituting Mn with Cr were revealed by modeling studies: it reduces the heat capacity of the materials up to 10% and it enhances the solar-to-fuel efficiency with operations near isothermal conditions because Cr substitution favors oxidation reaction at a higher temperature. Nevertheless, the reduction extent is low, which results in a lower efficiency compared to ceria. In the same family of manganite perovskites, the thermochemical activity of a large range of materials with different metal insertions in the A and B sites was characterized [69,148]. This experimental screening allows the correlation of the metal insertion with the redox activity of perovskites. From all compositions synthesized, (La,Sr)(Mn,Mg)O_3_ exhibits the best performance with a high reduction extent, a high fuel production level, and good thermal stability. Ca- and Al-doped SmMnO_3_ was investigated [149], showing a high CO production yield of 595 µmol/g (average) with Sm_0.6_Ca_0.4_Mn_0.8_Al_0.2_O_3_ when swinging between 1350 °C and 1100 °C. This high CO yield is attributed, according to a kinetic study, to a bulk diffusion reaction mechanism instead of a surface mechanism in the undoped SmMnO_3_. Moreover, this material possesses a high solar absorptance of 86.5% that eases the solar heating. Sr-doped SmMnO_3_ and Ca- and Ga-doped LaMnO_3_ perovskites for near-isothermal thermochemical CO_2_-to-fuel conversion were also proposed with similar outcomes [150,151]. 

The CaTi_0.5_Mn_0.5_O_3−δ_ perovskite was tested for two-step thermochemical cycling [152]. The combination of its intermediate enthalpy, ranging between 200 and 280 kJ (mol-O)^−1^, and large entropy, ranging between 120 and 180 J (mol-O)^−1^ K^−1^, creates favorable conditions for water splitting. A hydrogen yield of 10.0 mL g^−1^ was achieved in a cycle between 1350 °C (reduction) and 1150 °C (water splitting) and a total cycle time of 1.5 h. In similar work, the cubic perovskite SrTi_0.5_Mn_0.5_O_3−δ_ was also investigated [153], showing an attractive combination of moderate enthalpy, 200−250 kJ (mol-O)^−1^, and high entropy, with unusual δ dependence. Using a water-splitting cycle in which the material was thermally reduced at 1350 °C (with p_O_2__ ∼ 10^−5^ atm) and subsequently exposed to steam at 1100 °C (with p_H2O_ = 0.4 atm), a hydrogen yield of 7.4 mL g^−1^ was obtained with a stable H_2_/O_2_ ratio of 2.

More general studies dealt with the computational screening of perovskites for thermochemical applications [35,154,155,156], based on DFT calculations, as a time-saving methodology to discover new materials and appropriate compositions for thermochemical splitting cycles, but to date, these theoretical studies have not led to the discovery of a material that overpasses state-of-the art performance.

### 2.3. Dual-Phase Materials, Membranes, and High-Entropy Oxides

To increase the fuel production during two-step cycles, composite materials combining ceria and perovskite were explored. The oxygen exchange and CO_2_ splitting with the dual-phase material La_0.65_Sr_0.35_MnO_3_/CeO_2_ were investigated via thermochemical redox reactions [157], showing a synergetic effect that improves the reduction rate and the total CO production. From the XRD and Raman analysis results, the proposed mechanism to explain this behavior is the transport of oxygen from perovskite to ceria that acts as an oxygen diffusion channel. On the other hand, this mechanism has an adverse effect on the CO_2_ dissociation rate. The authors conclude that dual-phase materials combining ceria and perovskites should be deeply investigated and optimized to enable a fuel production improvement by using the synergetic effect of oxygen exchange. 

Another study focused on the thermochemical performance assessment of a new type of reactive composite material in a solar reactor: reticulated ceria foam covered with uniform thin perovskite coating, thereby forming a dual-phase layered heterostructure [115]. The La_0.5_Sr_0.5_Mn_0.9_Mg_0.1_O_3_ perovskite coating improved the reduction extent reached by the reactive material, with a significant increase of oxygen exchange in the dual-phase composite material during the reduction step compared to individual oxide components. The enhanced extent of reduction also showed a beneficial effect on the total amount of fuel produced by CO_2_ and H_2_O splitting. The study underlined the beneficial effect of composite redox materials (perovskite-coated ceria foams) in promoting the oxygen exchange capacity of ceria for solar thermochemical fuel production.

Ceria membranes coated with perovskite were investigated on-sun under various operating conditions [158], showing improved CO_2_ dissociation rates. Membranes coated with thin perovskite layers featuring enhanced ionic conductivity and oxygen partial pressure gradient enable isothermal CO_2_ splitting (1450–1550 °C) with high dissociation rates in comparison with pristine ceria membranes [159,160]. On one side of the membrane (feed side), flowing CO_2_ was dissociated and on the other side (sweep side), the oxygen release was favored through an inert gas sweeping. Mixed ionic–electronic conducting (MIEC) membranes for the thermochemical reduction of CO_2_ have been developed and are further reviewed in [161]. The membrane reactor is a process intensification technology in which both reaction steps are performed simultaneously under isothermal conditions with in situ product separation and the continuous production of O_2_ and CO (H_2_) on both sides of the membrane.

High-entropy oxides (HEOs), first published in 2015 [162], are a class of materials with a single phase containing five or more cations with a predominant part of the configuration entropy in front of the enthalpy contribution, leading to a negative Gibbs free energy. These oxides can mix, in a single crystallographic phase, metallic cations that could not be mixed without this predominant entropy contribution. These multicomponent oxides show great versatility with a wide range of structures and compositions. This new approach has created opportunities for the design and discovery of materials and is a promising way for many applications such as energy storage, catalysis, or optic applications [163].

Regarding thermochemical cycles, new compositions of high-entropy oxides could improve fuel production by increasing the reduction yield and the reactivity with oxidants. Since there are no theoretical models yet that could predict the structure/property relationship, current research on thermochemical fuel production with HEOs is led by cation choice and by experimentally characterizing their performance during thermochemical cycles. Polycation oxides (FeMgCoNi)O_x_ were tested for thermochemical water splitting [164] with a H_2_ yield of 10.1 mL/g and 1.4 mL/g with thermal reduction temperatures of 1300 °C and 1100 °C, respectively. The material performance at low temperature (≤1100 °C) overpassed the thermodynamic limits of well-known materials such as spinel ferrites or ceria. X-ray absorption characterization highlighted that Fe was the only redox-active species. The understanding of why the Fe^3+^ cation reduction extent was improved compared to ferrites and other iron-containing compounds is not well established and needs further investigation. Recently, different formulations of HEOs were investigated and compared using thermogravimetric analysis to evaluate their redox activity and ability to split CO_2_ during thermochemical cycles, and a maximum specific fuel output of 154 µmol/g per cycle was obtained with Fe_0.25_Mg_0.25_Co_0.25_Ni_0.25_O_x_ [165].

In other work on HEO for thermochemical hydrogen production, the thermal reduction was performed under microwave irradiation [166]. (FeMgCoNi)O_x_ was coated on a SiC foam and exposed to low-energy microwaves during brief irradiation. A maximum H_2_ yield of 122 mL/g was reached with a very short reduction time of 4 min under a 700 W irradiation followed by the water-splitting step. The mean value of H_2_ production was 63.47 mL/g over six consecutive splitting cycles. The authors also state that energy consumption using microwaves instead of concentrated solar energy could reduce the energy consumption by 97%, but the primary source (solar) would have to be converted into electricity, which induces low conversion efficiency compared to a direct use of concentrated solar energy. This very high production yield and fast reduction rate outperformed the existing performance of thermochemical H_2_ production, but suffers from material deactivation during cycling. In a subsequent study, Zr was inserted into the material [167], resulting in a H_2_ yield of 4.84 mmol/g with FeMgCoNiO_x_/Zr_0.6_ (with a reduction step temperature of 600 °C and a microwave power of 700 W). A new class of compositionally complex perovskite oxides (La_0.8_Sr_0.2_)(Mn_(1−x)/3_Fe_(1−x)/3_Co_x_Al_(1−x)/3_)O_3_ with new non-equimolar designs was explored [72] and a maximum H_2_ production of 89.97 mmol mol_oxide_^−1^ in a short 1 h redox duration was reported. Under the US project “HydroGEN: Advanced water splitting materials”, new high-entropy perovskite oxides (HEPOs) were investigated [168] with a H_2_ yield target of 400 µmol/g operating for 50 cycles of one hour (reduction and oxidation). Theoretical calculations (DFT) were realized to identify materials with an increased reduction entropy and a reduced vacancy formation enthalpy. This new and potentially transformative class of water-splitting materials presents a vast compositional space and an extreme tunability, enabling new design strategies. However, such computational methods are not suitable for providing insights into kinetic aspects of redox reactions, which is a key criterion for a material’s suitability in two-step cycles.

In summary, the above studies showed the tremendously high number of possibilities for material formulations as potential candidates for H_2_O- and CO_2_-splitting cycles, which may also represent a strong obstacle when attempting to select a suitable material for large-scale implementation. This explains why ceria currently remains the most favorable material for two-step cycles, given the simplicity of processing and, above all, the favorable fuel production kinetics. Moreover, the reported fuel production performance data are very broad for a large number of complex or exotic materials, which are also highly dependent on the synthesis methods and cycling conditions. Finally, several studies often omit investigating the kinetic rates of fuel production and report unusual, unexpected, or abnormal data regarding, e.g., the applied operating conditions or the fuel yields, which are difficult to reproduce and strongly require experimental validation and confirmation.

## 3. Ammonia Synthesis via Metal Oxide/Metal Nitride Chemical-Looping Cycles

Among the solar thermochemical processes to be developed, ammonia production is a promising way to decarbonate a huge part of the industrial carbon emissions. Ammonia is also considered to be an effective medium for hydrogen storage [169]. The conventional production process of ammonia (Haber–Bosch process) relies on fossil natural gas used to produce hydrogen via steam methane reforming (SMR), which leads to 2.6 metric tons of greenhouse gas (GHG) emissions per metric ton of ammonia produced. It represents about 2% of the world’s consumption of fossil fuels and is responsible for 1.2% of the total anthropogenic GHG emissions as it generates over 420 million tons of CO_2_ annually [170]. 

Over 90% of the global ammonia consumption, the second largest synthetic chemical product (after sulfuric acid), is manufactured from N_2_ and H_2_ via the catalytic Haber–Bosch process (N_2(g)_ + 3 H_2(g)_ ⇌ 2 NH_3(g)_). The reaction is exothermic (ΔH° = −91.9 kJ mol^−1^) and should thus occur spontaneously, however a significant energy input is required for nitrogen to reach the activated state due to its high dissociation energy (941 kJ mol^−1^). Using catalysts (e.g., iron-based catalyst) is necessary to lower the activation energy and to carry out the reaction in the operating conditions range of 400–650 °C and 20–40 MPa. Because of unfavorable thermodynamics, the yield is low even with added catalysts. At 30 MPa, the ammonia yield after one pass usually does not exceed 25%, thus requiring separation by condensation and recycling of the unreacted H_2_-N_2_ mixture. Moreover, the global process is characterized by a high energy consumption associated with the production of the N_2_ and H_2_ reactants. Commonly, H_2_ is produced by SMR, while N_2_ is obtained by cryogenic separation from air. Both of these processes require a large energy input, in the form of either high-temperature process heat or electricity, causing significant concomitant GHG emissions derived from fossil fuel combustion for heat and electricity generation.

To reduce its carbon intensity, several alternative pathways aim to consider green ammonia, a route to ammonia synthesis in which H_2_ derived from water electrolysis powered by alternative renewable energies replaces hydrocarbon-based H_2_, making NH_3_ production virtually CO_2_-free [170]. Efficient electrocatalysts for N_2_ fixation have been developed [171]. Other approaches are also considering carbon capture and storage (CCS) to minimize the carbon footprint of producing conventional ammonia (this route is referred as blue ammonia).

Ammonia has a higher volumetric energy density (12.7 MJ/L) than liquid hydrogen (8.5 MJ/L). Ammonia further offers high hydrogen storage densities as a liquid under mild pressurization and cryogenic constraints. The main interests of ammonia are the favorable storage in liquid form (NH_3_ boiling point = −33.4 °C at 1 atm versus −253 °C for H_2_), while containing 1.7 times more hydrogen per cubic meter (121 kg-H_2_/m^3^) than liquefied hydrogen (70.8 kg-H_2_/m^3^) [169], and ammonia, though hazardous to handle, is much less flammable than hydrogen. Furthermore, due to ammonia being extensively used for fertilizers in agriculture, a large ammonia infrastructure is already available. About 180 million metric tons of NH_3_ are produced annually worldwide, and 120 ports are also equipped with ammonia terminals. However, NH_3_ is currently produced almost exclusively from fossil fuels, which causes roughly over 1% of total GHG emissions, and represents about 20% of industrial natural gas demand and 5% of industrial coal demand.

**Figure 8:**
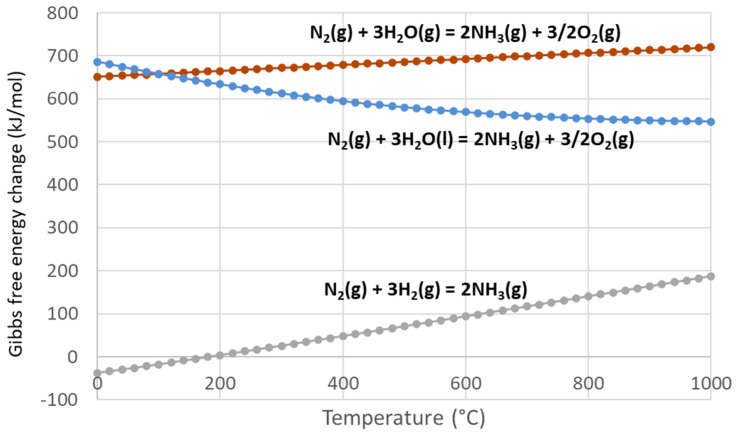
Variation of Gibbs free energy as a function of temperature for the ammonia synthesis reactions.

Alternative and promising mild condition ammonia production methods have been proposed including solid state synthesis, molten salt synthesis, thermochemical looping, and photocatalytic routes [172]. In particular, direct ammonia production from H_2_O and N_2_ without the intermediate H_2_ production step is currently being researched [173]. Ammonia could be considered as an energy carrier and could be a way to store concentrated solar energy into a chemical compound. Two-step solar thermochemical ammonia production involves redox pairs of metal oxides and metal nitrides being cycled for ammonia synthesis from water and nitrogen. The first endothermic step consists of converting the metal oxide into a metal nitride with N_2_ (M_x’_O_y’_ + N_2_ → M_x_N_y_ + ^3^/_2_O_2_). During this step, high temperatures are needed and can be provided by concentrated solar energy with or without the help of a reducing agent (e.g., C, CH_4_, biomass). During the second exothermic step, the metal nitride reacts with water vapor to produce ammonia and the initial metal oxide (M_x_N_y_ + 3H_2_O → 2NH_3_ + M_x’_O_y’_). The main advantage of this process is the ammonia production without any hydrogen consumption or electricity supply. The global reaction is highly endothermal (N_2_ + 3H_2_O → 2NH_3_ + ^3^/_2_O_2_, ΔH° = 633.6 kJ/mol) and thus can benefit from the use of solar energy as process heat input. Such a scheme can also sometimes be split into three steps with the same overall reaction: (1) nitridation: M (metal) + N_2_ → M_x_N_y_ (nitride), (2) hydrolysis: M_x_N_y_ + 3 H_2_O → 2 NH_3_ + M_x’_O_y’_ (metal oxide), and (3) solar reduction: M_x’_O_y’_ → M (regeneration) + ^3^/_2_O_2_. Figure 8 shows that the reaction of N_2_ with H_2_O cannot occur spontaneously and requires an energy input (heat or electricity).

Relative to the conventional production of NH_3_ via the Haber–Bosch process, the solar-driven process based on N_2_ and H_2_O feedstocks offers the following noteworthy advantages: (i) it eliminates the need for high pressures, minimizing process costs and safety concerns; (ii) it eliminates the need for catalysts, minimizing costs associated with their production and recycling; and (iii) it eliminates the need for H_2_ as feedstock, reducing energy consumption and associated CO_2_ emissions. However, it does not eliminate the need for nitrogen (N_2_ can be obtained via solar thermochemical air separation, as described in Section 5). Furthermore, using concentrated solar energy as the high-temperature process heat source for the endothermic reduction significantly reduces or completely eliminates concomitant CO_2_ emissions.

An alternative looping scheme was also proposed consisting of N_2_ reduction with a looped metal nitride (M_a_N_b−δ_ + δ/2 N_2_ → M_a_N_b_), followed by the separate hydrogenation of the lattice nitrogen into ammonia (M_a_N_b_ + 3δ/2 H_2_ → M_a_N_b−δ_ + δ NH_3_) [174]. A typical example is Co_3_Mo_3_N that yields Co_6_Mo_6_N and up to 8 mol% of its lattice nitrogen in the form of NH_3_ when reacted with H_2_ for 60 min at 400 °C [175]. However, the global reaction (N_2_ + 3H_2_ → 2NH_3_) is exothermal (and, thus, unfavorable for solar energy upgrading) and still requires the use of H_2_ like in the conventional process.

The ammonia production via a two-step cyclic process was studied, consisting of an endothermic carboreduction of Al_2_O_3_ in a N_2_ atmosphere to generate AlN, followed by the exothermic steam-hydrolysis of AlN to produce NH_3_ and to reform Al_2_O_3_ [176]. Both reaction steps proceed at 1 bar, without any added catalysts, and further bypass the energy-intensive production of hydrogen, resulting in significant fuel and cost savings. Preliminary environmental and economic analyses indicated favorable fuel economy and, hence, cost [177].

**Figure 9:**
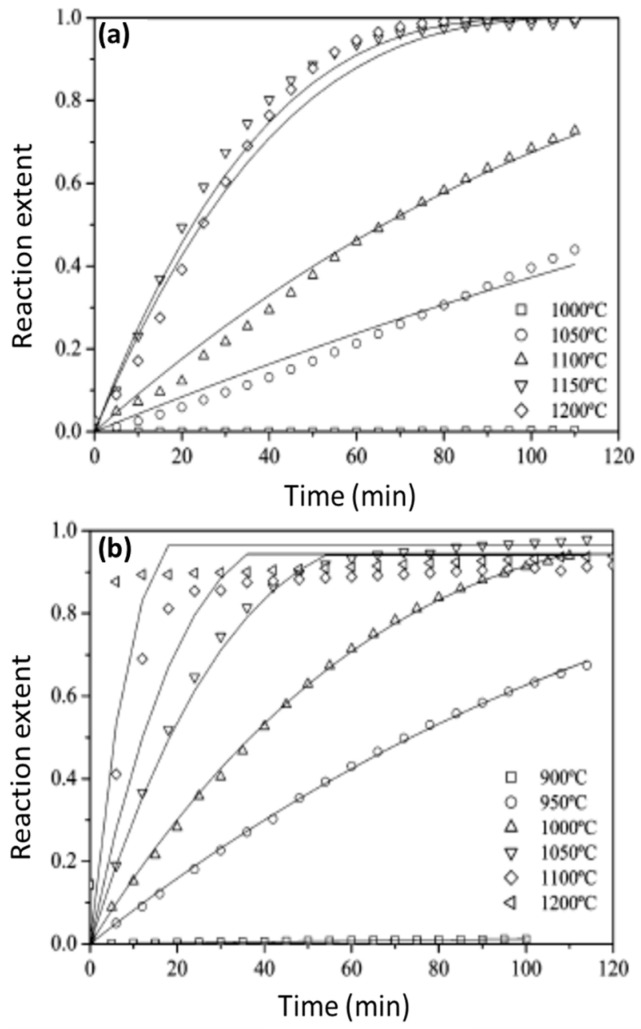
Reaction extent of AlN hydrolysis from 900 to 1200 °C [176] with (**a**) 10% H_2_O-Ar and (**b**) 80% H_2_O-Ar.

The chemical kinetics of both cycle steps were experimentally investigated by thermogravimetry. The carbothermal reduction of Al_2_O_3_ with activated carbon was investigated in the 1500–1700 °C range, yielding a reaction extent exceeding 80% after 30 min at 1700 °C. The AlN hydrolysis was investigated in the 900–1200 °C range with steam concentrations of 20, 40, 60, and 80%. The reaction proceeded at reasonable rates above 900 °C, and was completed after 120 min with 80% H_2_O-Ar (Figure 9, [176]). However, the yield of NH_3_ decreased above 1000 °C, because of the thermodynamically favorable NH_3_ dissociation at high temperatures, and was at its maximum (88%) at 1000 °C. The influence of the carbon source (namely, wood charcoal, petroleum coke, carbon black, and activated carbon) on the kinetics of the carbothermal reduction of Al_2_O_3_ was investigated in the 1500–1700 °C range, and the cyclability of the proposed process was determined by carrying out four subsequent reduction/hydrolysis cycles [178].

Experimental studies carried out with solar furnace addressed the carbothermal reductions of Al_2_O_3_, SiO_2_, TiO_2_, and ZrO_2_ with C in a N_2_ atmosphere to form AlN, Si_3_N_4_, TiN, and ZrN, respectively [179], confirming the possible synthesis of nitrides from their oxides at high temperatures with addition of a reducing agent.

Ammonia synthesis using the hydrolysis of lithium nitride was tested as well as the room temperature nitridation of lithium metal. The hydrolysis process of nitride, i.e., the reaction between pure Li_3_N and H_2_O, was performed under an inert atmosphere with a good reaction yield at 80 °C using water vapor [180].

The solar-driven thermochemical cycle for NH_3_ synthesis at near atmospheric pressure using a transition metal reactant and a Fresnel lens solar furnace was reported, consisting of reacting Cr metal powder with gaseous N_2_ to form Cr nitride, then hydrolyzing Cr nitride powder with steam to synthesize NH_3_ and Cr_2_O_3_, and finally reducing Cr_2_O_3_ powder back to Cr metal with mixtures of H_2_, CO, and N_2_ by heating to 1200–1600 °C. At about 1000 °C, it was shown that Cr readily fixed N_2_ as Cr nitride. The corrosion of Cr nitride with steam at 1000 °C and about 1 bar formed Cr_2_O_3_ and CrO while liberating only NH_3_ traces during the Cr nitride hydrolysis due to kinetic limitation [181].

The process conditions required to produce NH_3_ were achieved via Mo_2_N oxidation with H_2_O at around 500 °C and nitride recycling from the formed MoO_2_ with a N_2_/CO mixture at 750 °C [182]. Alternatively, NH_3_ can be synthesized via Mn_5_N_2_ oxidation with H_2_O above 500 °C and recycling from the formed MnO with a N_2_/H_2_ mixture at 1000–1230 °C. Furthermore, manganese nitride was identified as an ideal starting candidate material for the development of ternary metal nitride redox materials such as manganese nitride doped with iron.

In addition, to identify promising candidates for metal nitride/metal oxide pairs, high-throughput thermodynamic screening and electronic structure computations were also performed [183,184].

In summary, solar thermochemical nitride looping offers an alternative route to ammonia production, which operates under milder conditions and therefore has lower overall energy requirements than the Haber–Bosch process. A key advantage is that it directly converts water into ammonia without the intermediate hydrogen production step. The development of active nitride materials for the hydrolysis step to synthesize NH_3_ with high yields and the high-temperature regeneration in solar reactors for the nitridation step are the main key challenges for process performance increase and industrial applications.

## 4. Thermochemical Energy Storage at High Temperature

**Figure 10:**
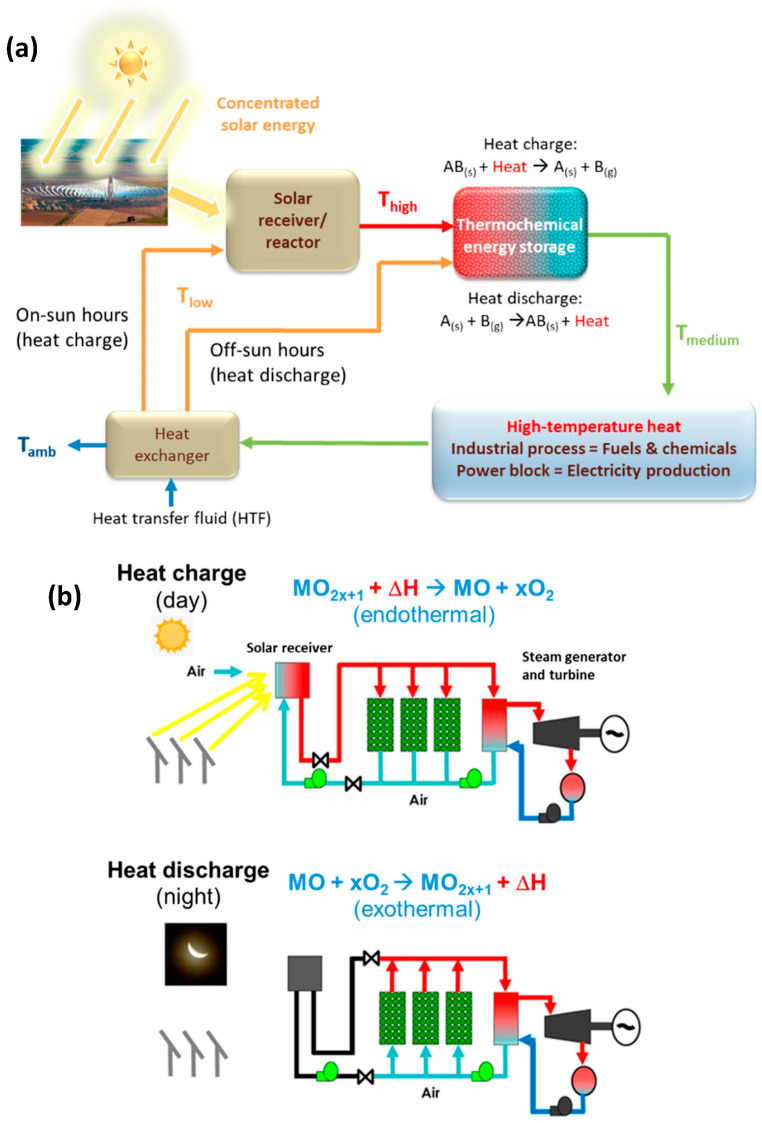
Thermochemical energy storage: (**a**) principle of the energy storage concept for supplying high-temperature process heat aimed at fuels and chemicals or electricity production; (**b**) application of thermochemical energy storage to concentrating solar power plants.

Being an intermittent and variable renewable energy, solar energy storage in the form of heat is a key issue. Intensive research and development are thus dedicated to identifying solutions for sensible, latent, and thermochemical heat storage to overcome the intermittency of sunlight for continuous solar process operations. The thermochemical energy storage (TCES) of solar energy at high temperatures can be performed by the means of reversible solid–gas reactions: AB(s) + ΔH ⇄ A(s) + B(g) [185,186,187,188,189,190,191]. This type of thermal energy storage can be associated with concentrating solar thermal power plants for continuous electricity generation (concentrated solar power, CSP [192]), or more generally to any type of industrial processes requiring high-temperature process heat (e.g., chemical industry, cement production, metallurgy, thermochemical fuel production). The main interest of such storage is the possibility to operate the solar process continuously by overcoming the inherent limitations of solar energy (intermittency and solar resource variability) [193]. The thermal effect of endothermal/exothermal reactions can be exploited provided that the reaction is reversible. The energy is stored in the form of chemical bonds in an amount equivalent to the enthalpy change of the reaction. The storage step (heat charge) corresponds to the solid material decomposition (endothermal step) that is carried out with the supply of solar energy [194]. The energy release step (heat discharge) corresponds to the reverse reaction to recover the energy stored as chemical bonds in the solid. This energy can be transferred to a heat transfer fluid (HTF) that can be then used to operate a thermodynamic cycle for electricity generation or directly fed to an industrial process for external energy supply (Figure 10). For instance, the utilization of redox pairs of oxides or mixed oxides (MO_2x+1_ + ΔH ⇄ MO + xO_2_) allows the operation of cycles under air in an open loop [195]. The main challenges in this field are related to the selection of the most suitable thermochemical systems depending on the downstream process requirements. 

TCES systems exhibit different energy storage properties including temperatures of the heat charge/discharge, heat storage capacity (energy storage density), reaction reversibility, and kinetics for both steps. In addition, suitable solar reactor concepts for the solid-gas reactions need to be designed, as well as the heat exchangers to transfer the solar energy to the reactive storage materials (solar receiver) and then to the heat transfer fluid. 

Studies on the state-of-the-art, screening, and selection of thermochemical reactions and candidate materials with high potential for energy storage at high temperatures (400–1200 °C) have been conducted [196,197]. Most TCES systems have only been assessed and tested at the laboratory scale so far. The energy storage density of such thermochemical pathways is usually 5- to 10-fold higher in comparison with sensible and latent energy storage systems. TCES systems based on reversible solid–gas reactions thus appear to be the most promising candidates for the long-term stable storage of solar energy. Accordingly, the reaction products can be stored at room temperature without energy losses during their storage and, therefore, the storage duration and transportation distance are theoretically unlimited. Regarding the key characteristics targeted when developing such systems, the process must be reversible with a constant conversion without performance losses during cycling to avoid a decrease in the material storage capacity. Another challenge is the optimization of the temperature gap between the charge/discharge steps that should be lowered to improve the process efficiency and facilitate reaction control.

TCES systems involving metal oxides (e.g., Co_3_O_4_/CoO, Mn_2_O_3_/Mn_3_O_4_, BaO_2_/BaO, CuO/Cu_2_O, Fe_2_O_3_/Fe_3_O_4_) are particularly attractive for CSP applications because air can be directly used as the heat transfer fluid in an open loop [198,199,200,201,202,203,204,205,206,207,208,209]. A critical review of thermochemical energy storage systems based on cobalt, manganese, and copper oxides was proposed [210]. The tuning of the redox properties (transition temperatures, oxygen storage capacity, reaction reversibility, etc.) is made possible via the synthesis of mixed oxides [211,212,213]. Accordingly, the reversibility and cycling stability of the Mn_2_O_3_/Mn_3_O_4_ system can be noticeably improved via the addition of Fe with a content higher than 15 mol% [211], as well as Co or Cu beyond 30 mol% [211,212,213]. It was demonstrated that the Fe incorporation (which is also an abundant material) into manganese oxide improves noticeably the redox performance of this system as it increases the heat storage density, narrows the thermal hysteresis between redox steps, and stabilizes and enhances the oxidation rate over long-term cycling operation [214]. The effect of Mn oxides co-doping with Fe and Cu on the temperatures of both forward and reverse redox reactions was evidenced, and it was shown that the addition of a certain amount of both dopants narrowed the thermal hysteresis of such redox reactions, while stable reversibility was achieved [215]. The capacity of both pure (Mn_2_O_3_ and Co_3_O_4_) and mixed oxides (Co_x_Mn_3−x_O_4_) to withstand several charge–discharge cycles was investigated, showing enhanced cyclability for the mixed oxides with low Mn content (x > 2.94) [216]. 

The Co_3_O_4_/CoO reversible system also offers noteworthy performance as a single oxide material [198,199,201,209]. Similarly, a thermochemical energy storage cycle based on the CuO/Cu_2_O redox couple was evaluated, yielding a gravimetric energy storage density associated with the endothermic/exothermic reversible reactions in the range of 470–615 kJ/kg for 50 to 65 wt% CuO−YSZ granules [217]. When considering binary oxides, Cu addition to Co_3_O_4_ enables to maintain a good cycling capability and high enthalpies at the expense of increasing sintering, while Fe addition to Co_3_O_4_ impacts its performance such as reaction enthalpy [211]. The energy storage density of oxides is correlated to the oxygen storage capacity [213]. Iron-doped cobalt oxides react at a similar temperature as pure cobalt oxide, but the reaction enthalpy gradually declines with increasing iron content [218]. The microstructural stability and the related long-term reversibility of chemical reactions, however, are higher with respect to pure cobalt oxide. Compositions with around 10% iron oxide were identified as offering appropriate enthalpies and being attractive in terms of microstructural stability. In a study dedicated to manganese–iron binary oxide, a specific reaction enthalpy of 271 J/g was obtained for a (Mn_0.75_Fe_0.25_)_2_O_3_ storage material and this material was further cycled in a packed-bed tube reactor [219,220]. The (Mn_0.8_Fe_0.2_)_2_O_3_/(Mn_0.8_Fe_0.2_)_3_O_4_ redox pair was selected as a promising system owing to its reduced cost, adequate thermodynamic characteristics, and excellent stability over prolonged cycling. Such redox materials could withstand over 75 reduction/oxidation (charge/discharge) chemical cycles and thus exhibited outstanding durability, which prompted a comprehensive assessment of the redox kinetics of both charging and discharging reactions [221]. Two Mn-based redox systems were also tested including the (0.8)(Mn_2_O_3_)/(0.2)(Fe_2_O_3_) composition due to its highest reaction enthalpy, and Ca-Mn based perovskites of stoichiometry CaMn_1−x_B_x_O_3−δ_ (with B being Mg, Ti and Al) [222].

In addition, a bench-scale demonstration of TCES using the magnesium–manganese oxide redox system was performed, yielding an average energy storage density of 2428 ± 469 MJ/m^3^ [223]. 

Other attractive oxide candidates for TCES have been studied, such as perovskites (ABO_3_ structure) featuring continuous redox activity over a variety of temperatures and oxygen partial pressures, thanks to vacancies that facilitate oxide ion transport [224,225]. As opposed to the oxides with discrete transitions in the metal oxidation state during redox reactions, perovskites offer continuous oxygen mobility through non-stoichiometric reactions and thus can adapt to various operating temperatures, which is convenient for high-temperature applications (>1000 °C). The structural and compositional flexibility of perovskite oxides along with their complex, yet tunable, redox properties offer unique optimization opportunities for TCES. A combinatorial approach was reported for the accelerated development and optimization of perovskite oxides for TCES application, based on density functional theory (DFT) simulation of over 2000 A/B-site doped SrFeO_3−δ_ [226]. The experimental results support the effectiveness of the high-throughput approach in determining both the oxygen exchange capacity and the oxidation enthalpy of the selected perovskite oxides. Several of the screened materials showed promising performance: Sr_0.875_Ba_0.125_FeO_3−δ_ exhibited a chemical energy storage density of 85 kJ kg^−1^ under air between 400 and 800 °C, whereas Sr_0.125_Ca_0.875_Fe_0.25_Mn_0.75_O_3−δ_ demonstrated an energy storage density of 157 kJ kg^−1^ between 400 °C (at 0.2 atm O_2_) and 1100 °C (at 0.01 atm O_2_). 

Ba-containing perovskite systems (BaCoO_3_, BaFeO_3_, and Ba_0.5_Sr_0.5_CoO_3_) exhibited large oxygen release ability under inert (p_O_2__ = 10^−6^ atm) or oxidizing atmospheres (p_O_2__ = 0.2 atm) up to 1050 °C, while only BaCoO_3_ could be fully re-oxidized at 600 °C in a 20% O_2_ atmosphere, with an energy storage capacity of 292 kJ/kg [224]. SrCoO_3−δ_, SrFeO_3−δ_, and SrMnO_3−δ_ were investigated and their oxidation enthalpy was measured (47.6 ± 5.7 kJ/kg, 81.7 ± 3.4 kJ/kg and 25.6 ± 5.8 kJ/kg, respectively) [227]. Regarding the compounds of Ba_1−x_Sr_x_CoO_3−δ_ [228], Ba content improves the redox capacities and reaction kinetics, while Sr content expands the reaction temperatures. Reversibility was maintained after 150 cycles, because the extended pores are beneficial to the oxygen diffusion. Ba_0.5_Sr_0.5_CoO_3−δ_ was suggested as an adequate TCES material with a reaction enthalpy of 202.20 kJ kg^−1^.

La_x_Sr_1−x_Co_y_Mn_1−y_O_3−δ_ (LSCM) and La_x_Sr_1−x_Co_y_Fe_1−y_O_3−δ_ (LSCF) are other possible candidates for high-temperature TCES due to their ability for cyclic endothermic reduction and exothermic oxidation [229]. Although the LSCF candidates reached a higher maximum δ, LSCM candidates showed a higher integrated reaction enthalpy due to the higher enthalpy per mole O_2_ at low δ. LSCM3791 perovskite demonstrated the highest weight-specific reaction enthalpy of 250 kJ/kg. La_x_Sr_1−x_(Mn, Fe, Co)O_3−δ_ and Ba_y_Sr_1−y_CoO_3−δ_ perovskite oxide powders were also investigated as potential TCES materials above 600 °C in terms of chemical reactivity, charging/discharging temperatures and heat storage capacities, oxygen uptake/release kinetics, and thermochemical cycling repeatability [230].

A redox cycle of calcium manganite for solar high-temperature TCES was proposed. CaMnO_3_ exhibits an energy storage capacity of 893.2 kJ/kg from 200 °C to 1000 °C, and a non-stoichiometry of 0.133 in N_2_ with excellent stability during 150 redox cycles between 500 °C and 1000 °C [231]. The redox cycling of doped CaMnO_3−δ_ represents an attractive route for cost-effective TCES at high temperatures. The role of dopants is mainly to improve the thermal stability and the global storage density of the material. Cobalt-doped CaMnO_3−δ_ perovskites were explored: CaCo_0.05_Mn_0.95_O_3−δ_ offered an enhanced redox capacity and a TCES density of ∼571 kJ kg^−1^ [232]. The impact of La and Fe doping on the thermochemical energy storage properties of CaMnO_3−δ_ was studied for tuning the thermochemical properties (i.e., reaction enthalpy and entropy, reaction extent, reduction onset temperature, and thermal stability) [233,234]. Both 10 and 30 cat% La (A-site doping) or Fe (B-site doping) stabilized the base oxide, preventing decomposition up to 1200 °C at p_O_2__ = 0.008 atm, and offering access to higher operating temperatures. The thermochemical energy storage capacity of Ca_0.9_La_0.1_MnO_3_ (~265 kJ/kg) and CaFe_0.1_Mn_0.9_O_3_ (~344 kJ/kg) was found to be comparable or slightly higher than that of undoped CaMnO_3_ (~272 kJ/kg).

CaAl_0.2_Mn_0.8_O_3−δ_ and CaTi_0.2_Mn_0.8_O_3−δ_ perovskites were developed for high-temperature TCES applications [235], e.g., in support of air Brayton solar power generation. Elemental substitution and doping of calcium manganite (with Al, Co, Fe, La, Sr, Ti, Y, Zn, and Zr) were considered to increase the reduction enthalpy [236]. 

The thermodynamics and kinetics of Ca_1−x_Sr_x_MnO_3−δ_ (with x = 0.05 and 0.1) particles were explored for TCES redox cycles in which the particles are reduced in N_2_ (p_O_2__ ~ 10^−4^ bar) during heating to high temperatures up to 1000 °C in a solid particle solar receiver. Both the chemical and sensible energies stored in the reduced perovskite particles are intended to be delivered to a power cycle via heat recovery from both the re-oxidation and cooling of the material. A specific TCES density of 620 kJ kg^−1^ was reached for Ca_0.95_Sr_0.05_MnO_3−δ_ and particles showed excellent phase stability for the desired redox conditions after 1000 redox cycles [237].

A two-step cycle was considered for solar TCES based on aluminum-doped calcium manganite (CaAl_0.2_Mn_0.8_O_3_) for direct integration into air Brayton cycles to produce electricity. A 5 kW(thermal) inclined granular-flow solar thermochemical reactor was manufactured and tested to examine the first cycle reduction step under continuous reactor operation with particle temperatures over 1073 K, despite an issue of particle agglomeration [238]. 

Alternatively, it must be noted that other types of materials have been proposed. For instance, TCES systems based on alkaline earth metal carbonates show a practical interest [225,239,240,241,242,243] and have been largely developed for post-combustion CO_2_ capture (e.g., Ca-looping cycles). The CaCO_3_/CaO system involving successive calcination/carbonation reactions is advantageous as the natural mineral resource is abundant, low cost, and offers a high energy storage potential. The loss-in-capacity due to sintering and pore blockage during cycling (carbonation step) can be overcome by using composite materials or stabilizing agents to maintain the system capacity during charge/discharge steps, as they usually undergo a drop of reactivity over multiple carbonation/calcination cycles due to sintering. Among the alkaline earth carbonates, CaCO_3_ and SrCO_3_ are the most suitable candidates for energy storage, with a high gravimetric energy storage density [244]. In contrast, BaCO_3_/BaO is more challenging due to a melting issue that impacts reaction reversibility. The addition of an inert material such as MgO or Al_2_O_3_ can be used to improve the stability of carbonates during cycles [239,245]. Natural minerals were also shown to be more resistant to deactivation. CaO derived from dolomite (dolime) was proved to exhibit a high multicycle conversion regardless of the particle size, which can be explained by the presence of inert MgO grains enabling the reacting gas to percolate inside the porous particles [246]. Finally, metal hydroxides (M(OH)_2_/MO) [225] and sulfates (MSO_4_/MO) also represent appropriate TCES systems due to their high energy storage density, at the expense of additional issues related to corrosive gas products. Such systems must also be operated in a closed loop to recycle the gas species (CO_2_, H_2_O, or SO_2_) and avoid their release out of the process. 

The integration of TCES systems to industrial processes is thus currently a great opportunity to supply solar heat on demand and warrant round-the-clock operation. Possible solar reactor concepts can be based either on the continuous-flow particles reactor or monolithic reactor concepts [42,247,248,249]. In such reactors, porous monolithic absorbers or particles play the role of the solar interface. The main benefits of the former are the high surface area, effective volumetric absorption and heat transfer, and straightforward integration of concentrated sunlight, while the advantages of the latter are the possible transport and storage of particles, performing as both heat transfer and storage medium at the same time, the requirement of particulate material in various industrial processes, and the possible tuning of mass flow rate as control parameter. 

**Figure 11:**
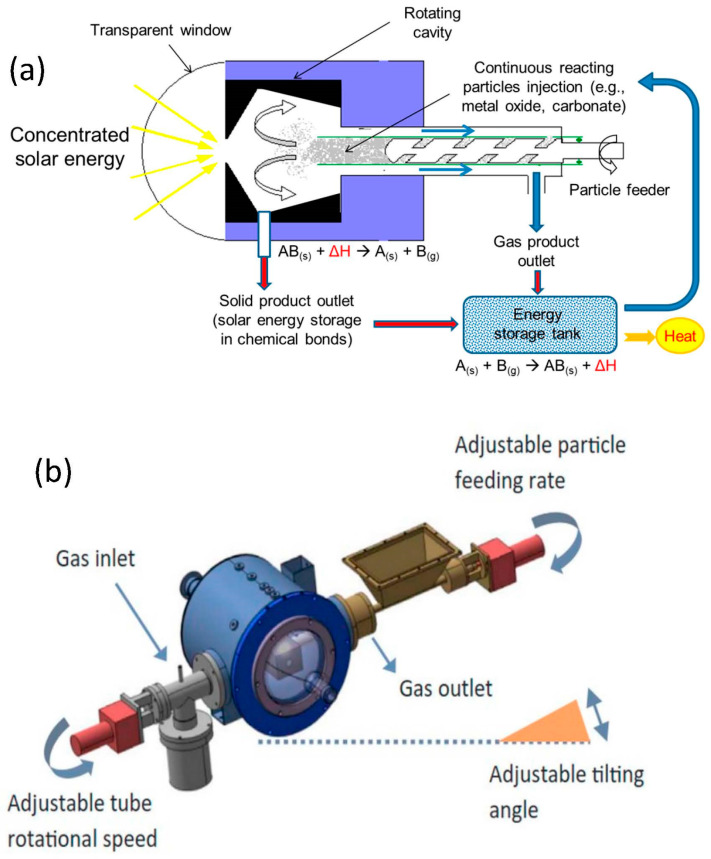
Operating principle of continuous-flow solar reactors featuring particle feeding in a rotary kiln applied to thermochemical energy storage under continuous operation. (**a**) Example of a windowed directly irradiated cavity-type solar reactor for effecting the reactions in a continuous mode and initially developed for continuous ZnO reduction. (**b**) Example of an indirectly irradiated solar reactor design based on a tilted rotary tube and equipped with a continuous particle feeding and collecting system [258].

Various solid–gas solar reactors were developed for particles processing or thermal decomposition processes around 1000 °C, which could be further implemented for TCES [248,250,251]. For example, continuous-flow reactor concepts for solar energy storage have been proposed and demonstrated, as illustrated in Figure 11. The reactors are generally composed of a cavity-type solar receiver for radiation absorption and heat transfer to the continuously injected reactive particles. Directly irradiated cavity-type solar reactors based on a rotary kiln can be suitable options for effecting the reactions in a continuous mode and were initially developed for continuous ZnO reduction [252,253,254]. The reacted particles can then be recovered at the reactor outlet in a storage tank for the discharge step, releasing heat (Figure 11a). Directly irradiated rotary kilns were investigated in a batch operation with regard to the on-sun reduction and off-sun oxidation of the redox pairs Co_3_O_4_/CoO [255] and CuO/Cu_2_O [256,257].

A novel rotary-tube solar reactor was also tested for continuous processing of reactive particles involved in high-temperature (500–1600 °C) thermochemical reactions [258] (Figure 11b). This type of reactor offers several advantages: (i) the indirect reactants heating thus avoiding product deposition on the optical window (due to the reacting zone being separated from the zone receiving solar irradiation), (ii) the continuous solid reactive particles injection, (iii) the tube rotation enabling facilitated bed particle transport and circulation to the outlet, (iv) the uniform reactive zone heating, (v) the direct contact between particles and the inner tube wall, enabling optimal heat transfer, and (vi) a long particle residence time controllable by adjusting the particle feeding rate, tube rotational speed, and tube tilting angle. 

Reactors based on monolithic structures are another option. The redox performance of several cobalt oxide-based structured bodies (flow-through pellets) was assessed in the temperature range of 800–1000 °C for energy storage in future CSP plants [259]. The most promising materials were found to be the cobalt oxide–alumina and cobalt oxide–iron oxide composites. An evaluation of small-scale honeycomb structures used as compact reactors/heat exchangers was performed by exploiting the cyclic heat storage redox scheme based on cobalt/cobaltous oxide (Co_3_O_4_/CoO) [260]. The considered structures were subjected to multi-cyclic redox operations under air flow, in the temperature range of 700–1000 °C. A pilot-scale redox-based TCES system was operated inside a solar power tower plant [261]. The storage unit was composed of inert honeycomb supports (cordierite) coated with redox active material (88 kg of cobalt oxide), and its storage capacity (47.0 kW h) was almost double that obtained for a sensible-only storage unit made of uncoated cordierite honeycombs (25.3 kW h).

The variety of candidate materials for thermochemical energy storage with relevant adaptable properties makes possible their combination with various industrial processes. In comparison with sensible and latent heat storage technologies, TCES is a more flexible approach offering a wide range of operating temperatures adaptable to various high-temperature processes, a high storage capacity without thermal losses during long periods including seasonal energy storage, and thus a strong potential for the development of concentrating solar applications.

## 5. Thermochemical Air Separation and Oxygen Pumping

**Figure 12:**
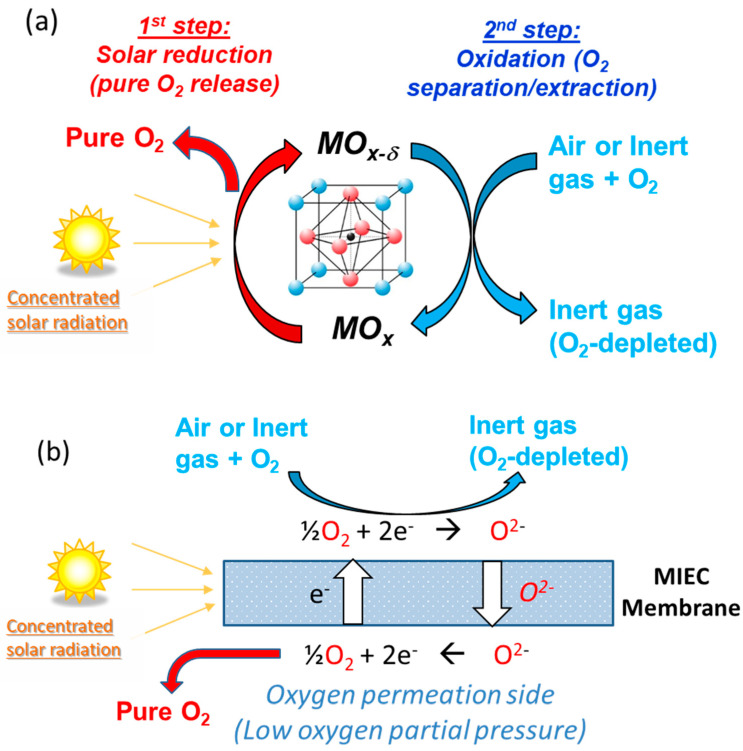
Thermochemical oxygen separation from air or oxygen pumping based on: (**a**) solar-driven two-step chemical looping for oxygen separation and release via temperature or pressure swing cycle; (**b**) direct oxygen separation and extraction via a ceramic oxygen-conducting membrane operating isothermally with partial pressure gradient for continuous oxygen transfer between the membrane sides.

This section addresses the metal oxide redox cycles for thermochemical oxygen separation from air and thermochemical oxygen pumping. The redox reactions are similar to those involved in TCES, but instead of exploiting the thermal effect of reactions, the oxygen storage capacity of the materials is of prime importance for the oxygen absorption. On the one hand, oxygen separation from air (also known as chemical looping air separation) aims to generate a pure flow of O_2_ (along with pure N_2_ that is necessary for ammonia synthesis, for example). On the other hand, thermochemical oxygen pumping targets the removal of oxygen traces from inert gas flows either to lower the O_2_ partial pressure in the flowing gas or to purify and recycle the inert gas contaminated with diluted O_2_ (released from the reduction step of the redox cycles). Thus, multiple applications can be considered including pure O_2_ and N_2_ generation, thermochemical pumping for oxygen partial pressure reduction, and inert gas cleaning/purification for recycling. The key challenges are designing robust oxygen sorbents with suitable redox properties and fast redox kinetics, while the characteristic of major importance is the oxygen storage capacity of the materials. The two-step process involving oxygen separation from a gas stream and pure O_2_ release is schemed in Figure 12a.

Thermochemical oxygen pumping was considered for improved fuel production in solar redox cycles [262,263,264]. In such cycles, a method for the optimization of the high-temperature reduction step is to remove the oxygen released during this step, in order to increase the reduction extent of the redox material. The two main options considered for oxygen removal are dilution using a sweep gas and vacuum pumping to lower the total pressure. Both methods result in lowering the oxygen partial pressure, which increases the driving force for the generation of oxygen vacancies in the oxide lattice. To reach the low oxygen partial pressures required, the oxygen removal results in significant energy penalties for the process, mainly related to sweep gas purification in the first case and pumping efficiencies at low pressures in the second case [264,265,266,267,268,269,270]. 

Thermochemical oxygen pumping is a promising option with large energy saving potential. It avoids the efficiency drop of mechanical pumps at low pressures related to the large volumetric flow rates. It involves the use of metal oxide redox materials acting as scavengers to absorb the oxygen contained in the gas flow. It makes use of a second redox material which is applied to absorb the oxygen released from the first splitting redox material. The thermodynamic requirements are different for the two redox materials, since the material used for oxygen pumping does not need to be able to split H_2_O or CO_2_. The pumping metal oxide can be reduced using waste heat and re-oxidized in a second step [264]. In contrast to materials for H_2_O or CO_2_-splitting cycles, the metal oxide for oxygen pumping directly absorbs oxygen in the re-oxidation step. Thus, the materials capable of splitting water or carbon dioxide differ from the materials used as pumping materials for oxygen absorption. The latter oxides generally allow reduction at significantly lower temperatures or higher oxygen partial pressures and with lower energy input, and as such, the materials used for pumping do not actually need to split H_2_O or CO_2_ oxidants, but absorb oxygen directly. 

The range of redox materials applicable for oxygen pumping is broad. Generally, such oxides that are readily reduced at low temperature under an ambient (air) atmosphere are among those also proposed for thermochemical energy storage. Co_3_O_4_/CoO-based oxygen pumping, for instance, can be an option. Nevertheless, the oxidation reaction kinetics may become a limiting factor for practical application, as those reactions involving a phase transition with re-arrangement of the crystal structure take several minutes even in air at high temperatures [198]. Relevant materials are expected to exhibit attractive thermodynamics as well as kinetics. Typically, suitable materials are based on perovskites and also have a higher oxygen storage capacity than ceria under the same conditions. For this reason, although being re-oxidized easily with oxygen, ceria is not a good candidate material for oxygen pumping, as the reduction temperature is too high to reach a sufficient oxygen sub-stoichiometry δ. 

When using perovskite-based redox materials, the lower limit of O_2_ partial pressures for solar thermochemical cycles from an energy demand perspective may be pushed well below 10^−10^ atm [262]. At low O_2_ partial pressures, thermochemical pumping can be much more efficient than mechanical pumping, and the efficiency can be further enhanced by recovering the heat released during the exothermal oxidation of the pumping material. For low O_2_ partial pressures (<1 mbar), the energy demand per removed mol of O_2_ for cycling a pumping material between the reduction temperature and the oxygen absorbing temperature can be typically several orders of magnitude lower than the corresponding energy demand of mechanical vacuum pumping [264]. 

Among the possible perovskites, different transition metals can be inserted in order to adjust the oxygen affinity of the reduced perovskite during the oxidation step, or the reduction temperature and O_2_ partial pressure. For such non-stoichiometric oxides, the oxygen storage capacity is not fixed (as in the case of stoichiometric oxides), but is dependent on both temperature and O_2_ partial pressure. It is possible to tune the redox properties of perovskite materials by tuning their composition. Theoretical/computational studies have been performed on (A’_x_A”_1−x_)(M’_y_M”_1−y_)O_3_ perovskites to calculate redox enthalpies and to predict the amount of O_2_ released [35]. The aim is to create a perovskite search engine for thermochemical applications. This can help the rapid design of suitable perovskites for air separation and oxygen pumping, for instance, and to identify ideally suitable materials for each range of targeted O_2_ partial pressures. A(Mn,Fe)O_3−δ_ perovskites containing an alkali earth metal on the A site are excellent materials for air separation and offer tunable oxygen storage capacities by adjusting the Fe content [271,272]. Fe-rich alkali earth metal perovskites are more suitable to reduce and can store more oxygen at lower pressures, whereas Mn-rich alkali earth metal perovskites have a higher oxygen affinity in the reduced state, thus enabling lower O_2_ partial pressures to be reached. Oxygen pumping in thermochemical cycles was demonstrated with SrFeO_3−δ_ as a pumping material [263]. The CaMnO_3_ and Ca_0.8_Sr_0.2_MnO_3_ perovskite oxides also exhibit a continuous non-stoichiometry over a range of temperatures and oxygen partial pressures. Ca_0.8_Sr_0.2_MnO_3_ shows a lower reduction enthalpy and thus can be more easily reduced, and it also exhibits faster kinetics, thus maintaining activity at lower temperatures [273]. Overall, Ca_0.8_Sr_0.2_MnO_3_ perovskite offers very promising properties for thermochemical redox applications, including a gravimetric oxygen storage up to 4% by weight, high stability, and rapid reversibility, with re-oxidation completed in less than 1 min at 400 °C.

The thermal swing reduction–oxidation cycle of Me(Ba, Ca, or Mg)SrCoCu perovskite compounds for oxygen separation from air was investigated to supply oxygen to oxyfuel energy systems [274]. The studied perovskites were Me_0.5_Sr_0.5_Co_0.8_Cu_0.2_O_3_ formulations, where the A-site substitution was achieved by different cations. During reduction and oxidation reactions, Ba and Ca substitutions in A-site resulted in the highest and lowest oxygen release, respectively. With respect to the real applications for oxygen separation from air, Ba substitution was proved to be preferable due to the short temperature swing cycles for the oxygen uptake and release. 

A screening study of materials revealed that the non-stoichiometric Sr_0.8_Ca_0.2_FeO_3−δ_ perovskite oxide is a promising candidate redox material, offering fast kinetics and a 75% higher gravimetric oxygen capacity compared to state-of-the-art SrFeO_3_ [275]. The impact of Ca^2+^ doping on oxygen ion diffusion in Sr_1−x_Ca_x_FeO_3−δ_ (with x = 0, 0.125, 0.25, 0.375, 0.5) was investigated by combining both density functional theory (DFT) calculations and experimental measurements [276]. The oxygen ion diffusion was determined by using two key factors including oxygen vacancy formation and migration. The DFT results showed that the energies of oxygen vacancy formation greatly decreased as Ca^2+^ content reached x = 0.125, gradually decreased with Ca^2+^ contents up to 0.375, and finally increased as the Ca^2+^ content reached x = 0.5. An appropriate Ca^2+^ doping of x = 0.125–0.375 promoted the oxygen ion diffusion in Sr_1−x_Ca_x_FeO_3−δ_. Strontium ferrite with B-site aluminum dopant molar fractions of 0 to 0.20 (SrAl_y_Fe_1−y_O_3−δ_) was considered and it was shown that aluminum doping increased the oxidation extent for sufficiently low O_2_ partial pressures, but also limited the non-stoichiometry range and thus the separation capacity [277]. 

High oxygen production from binary Cu-Co oxides was also achieved [278]. A high Co content in the presence of Cu (Co_0.8_Cu_0.2_O_x_) led to fast cycles of oxygen uptake and release and high oxygen production rates (∼5.0 wt%). The robustness of the binary Co-Cu oxides paves the way to oxygen production for the new generation of oxyfuel coal power plants. 

(Ca,Sr) ferrite and manganite perovskites were studied for thermochemical air separation due to their oxygen non-stoichiometry δ, which can be varied by modifying the temperature and O_2_ partial pressure [271]. SrFe_0.95_Cu_0.05_O_3−δ_ and Ca_0.8_Sr_0.2_MnO_3−δ_ perovskites showed the highest gravimetric oxygen storage capacity below 1200 °C. High reaction rates (reaction completed within seconds) and gravimetric oxygen storage capacities (over 2% in air) were reached. If high heating and cooling rates can be achieved, such perovskite materials can be used to separate large amounts of air per unit time and mass of oxides, solely using solar or waste heat as an energy source. Furthermore, the two identified materials could be processed in cascade with SrFe_0.95_Cu_0.05_O_3−δ_, removing most of the oxygen from air, followed by a further decrease in partial pressure using Ca_0.8_Sr_0.2_MnO_3−δ_.

Perovskites with multivalent transition metal oxide offer interesting oxygen storage capacity (OSC) properties due to their structural stability over a large amount of oxygen deficiency. Two series of Mn-containing perovskites with oxygen non-stoichiometry δ, namely Sr_x_Ca_1−x_Mn_0.75_Nb_0.25_O_3−δ_ and Sr_x_Ca_1−x_Mn_0.5_Nb_0.25_Zr_0.25_O_3−δ_, were cycled between Ar-5% H_2_ and oxygen [279].

Perovskite-structured Sr_1−x_Ca_x_Fe_1−y_Co_y_O_3_ oxygen sorbents were also investigated and demonstrated for oxygen production [280]. Cobalt doping improved oxygen productivity at 500 °C and stabilized the perovskite structure.

La_0.5_Ba_0.5_CoO_3−δ_, Pr_0.5_Ba_0.5_CoO_3−δ_, and Y_0.5_Ba_0.5_CoO_3−δ_ perovskite compounds were investigated using dynamic oxygen exchange measurements. Y_0.5_Ba_0.5_CoO_3−δ_ was identified as the most active for thermochemical oxygen separation and production. This material showed the fastest reaction rates, lowest reaction temperatures, and highest oxygen exchange capacities compared to the other tested perovskites and the state-of-the-art SrCoO_3_ and SrFeO_3_ materials [281].

Different perovskite compositions based on the (Ca,Sr)(Mn,Fe)O_3−δ_ system were synthesized, tested, and compared to cobalt oxide with respect to their capability of reducing the oxygen partial pressure in a reaction chamber where ceria thermal reduction is carried out [282]. A temperature swing for the pumping material significantly enhanced the amount of oxygen that the pumping material could capture from the splitting material. The best-performing formulations were CaMnO_3−δ_ and CaCr_0.1_Mn_0.9_O_3−δ_. Ca-Mn-based perovskites outperformed Co_3_O_4_ in oxygen pumping.

The oxygen storage capacity of Ca_2_(Al_x_Mn_1−x_)_2_O_5+δ_ (0.50 ≤ x ≤ 0.67) with a Brownmillerite-type structure was investigated [283]. This oxide can store and release in a highly reversible manner a large amount of excess oxygen (∼3.0 wt%) topotactically in response to variations in temperature and surrounding atmosphere composition. Remarkable capacity and responses of oxygen storage were only obtained near x = 0.50 (Ca_2_AlMnO_5+δ_), and rapidly deteriorated as the Al content increased.

SrFeO_3−δ_ and SrMn_0.1_Fe_0.9_O_3−δ_ materials showed rapid kinetics and stability over 1000 redox cycles [284]. The oxygen sorption and desorption properties of Sr–Co–Fe oxides were investigated [285,286]. The amount of sorbed oxygen from SrCo_0.85_Fe_0.15_O_3−δ_ was 11.7 cm^3^·g^–1^ at 300 °C, which was larger than that (8.6 cm^3^·g^–1^) for a benchmark oxygen sorbent (La_0.1_Sr_0.9_Co_0.9_Fe_0.1_O_3−δ_) [287]. SrCo_0.8_Fe_0.2_O_3−δ_ sorbent was considered for the high-temperature production of oxygen-enriched carbon dioxide stream [288]. SrFeO_3_ was studied [289] and selected for its relatively low reduction temperature and strong oxygen affinity. Rapid re-oxidation kinetics were achieved for SrFeO_3_ at temperatures as low as 300 °C. Multiple reduction and oxidation cycling experiments were carried out (with the oxidation step performed under both synthetic air and inert gas with 1% O_2_ concentration), demonstrating successful oxygen removal and no degradation of the material over multiple cycles. The design principles of perovskites for thermochemical oxygen separation were elaborated based on electronic structure calculations to predict the oxygen exchange capacity of perovskites, and SrCoO_3−δ_ was identified as an ideal material for the solar-driven thermochemical O_2_ separation [290]. Furthermore, high-purity nitrogen (N_2_) production from air by pressure-swing adsorption combined with SrFeO_3_ redox chemical-looping was also demonstrated [291].

This technology could offer a very attractive, reversible cycle for the removal of oxygen impurities from various gas streams, particularly in combination with conventional pressure-swing adsorption. Thermochemical air separation is another application with large energy saving potential (e.g., when compared with cryogenic distillation) as it provides pure streams of O_2_ (required in oxy-combustion technologies) and N_2_ (relevant source for ammonia synthesis). 

Noticeably, another approach consisting of using oxygen-conducting ceramic membranes made of various oxide formulations has also been widely considered for air separation [292,293,294,295,296,297,298,299,300,301,302,303] and could be further implemented with the supply of solar energy, as illustrated in Figure 12b. The oxygen separation can be performed continuously with the absorption of oxygen on the feed side and the permeation of oxygen ions in the oxide lattice to release pure oxygen on the opposite side, while operating isothermally without the need for alternating the temperature.

## 6. Conclusions

Thermochemical metal oxide cycles can be applied to different thermochemical applications relying on concentrated solar energy as the energy source for chemical-looping processing. The solar redox cycles for splitting H_2_O and CO_2_ are currently the most developed and a plethora of materials have been considered, including volatile and non-volatile as well as stoichiometric and non-stoichiometric reactions. The most common materials are based on simple oxides (volatile: ZnO/Zn, SnO_2_/SnO, non-volatile: Fe_3_O_4_/FeO, CeO_2_/Ce_2_O_3_) and non-stoichiometric redox systems (CeO_2_/CeO_2−δ_, ABO_3_/ABO_3−δ_). The former systems proceed via discrete transitions in the metal oxidation state accompanied by a phase change during the reduction step, whereas the latter proceed via the creation of oxygen vacancies in the crystalline lattice without any phase change. Such materials need to split an oxidizer and, thus, must possess adequate thermodynamic properties to overcome the thermodynamic barriers. They usually require high reduction temperatures (above ~1300–1400 °C under inert gas to ensure low-oxygen partial pressure) and a large temperature swing between the redox steps. In addition, kinetic considerations must be taken into account to fulfill the kinetic requirements of gas–solid reactions. Thus, the materials’ morphology and microstructure need to be tuned to facilitate the heat and mass transfer, as well as to promote the surface and bulk reactions. 

Regarding thermochemical energy storage and oxygen removal applications using reversible redox reactions, the constraints imposed on the material are much less stringent than for splitting cycles. Indeed, the material does not actually need to split an oxidizer (such as H_2_O or CO_2_), but to absorb and release oxygen directly in a reversible way, which is much easier and allows for a reduction at lower temperatures or at higher oxygen partial pressures (reactions are generally carried out under air) and with lower solar energy input (waste heat can be used). Typically, these materials exhibit a high oxygen storage capacity and include simple oxides (e.g., Co_3_O_4_/CoO, Mn_2_O_3_/Mn_3_O_4_, BaO_2_/BaO, CuO/Cu_2_O, Fe_2_O_3_/Fe_3_O_4_), mixed oxides, and perovskites (with formulations different from those used in splitting cycles). It is thus important to note that the materials used for thermochemical heat storage or oxygen gas separation cannot be used for the splitting cycles, and vice versa. 

As for ammonia synthesis via metal oxide/nitride chemical looping cycles, this research field is not yet widely developed and more research efforts are needed to demonstrate and further develop this promising approach, especially regarding the solar-driven nitride regeneration step at high temperatures. The novel process relies exclusively on the use of N_2_ and H_2_O instead of H_2_ for ammonia synthesis, and it could thus represent a promising alternative to the conventional processes. 

In addition to material formulations and chemical system reactivity, the development of suitable solar reactor technologies is also challenging for continuous solar chemical processing. Process scale-up, integration, and coupling with relevant concentrating solar systems must be considered for the different applications involving renewable fuel synthesis based on H_2_O and CO_2_ splitting, green ammonia synthesis, thermochemical storage of solar energy and oxygen pumping/separation.

## Data Availability

Data sharing is not applicable to this article.
